# Human venous valve disease caused by mutations in *FOXC2* and *GJC2*

**DOI:** 10.1084/jem.20160875

**Published:** 2017-08-07

**Authors:** Oliver Lyons, Prakash Saha, Christopher Seet, Adam Kuchta, Andrew Arnold, Steven Grover, Victoria Rashbrook, Amélie Sabine, Gema Vizcay-Barrena, Ash Patel, Francesca Ludwinski, Soundrie Padayachee, Tsutomu Kume, Brenda R. Kwak, Glen Brice, Sahar Mansour, Pia Ostergaard, Peter Mortimer, Steve Jeffery, Nigel Brown, Taija Makinen, Tatiana V. Petrova, Bijan Modarai, Alberto Smith

**Affiliations:** 1 Academic Department of Vascular Surgery, Cardiovascular Division, BHF Centre of Research Excellence, King’s College London, St Thomas’ Hospital, London, England, UK; 2 Center for Ultrastructural Imaging, King’s College London, London, England, UK; 3 Department of Ultrasonic Angiology, Guy’s and St Thomas’ NHS Foundation Trust, London, England, UK; 4 Division of Hemostasis and Thrombosis, Beth Israel Deaconess Medical Center, Boston, MA; 5 Harvard Medical School, Boston, MA; 6 Department of Fundamental Oncology, Ludwig Institute for Cancer Research, Zurich, Switzerland; 7 Division of Experimental Pathology, Centre Hospitalier Universitaire Vaudois, University of Lausanne, Epalinges, Switzerland; 8 Feinberg Cardiovascular Research Institute, Northwestern University School of Medicine, Evanston, IL; 9 Department of Pathology and Immunology, University of Geneva, Geneva, Switzerland; 10 South West Thames Regional Genetics Service, St George’s Hospital, London, England, UK; 11 Cardiovascular and Cell Sciences Institute, St George’s Hospital, London, England, UK; 12 Institute of Medical and Biomedical Education, St George’s Hospital, London, England, UK; 13 Rudbeck Laboratory, Department of Immunology, Genetics and Pathology, Uppsala University, Uppsala, Sweden

## Abstract

Venous valves (VVs) prevent venous hypertension and ulceration. We report that *FOXC2* and *GJC2* mutations are associated with reduced VV number and length. In mice, early VV formation is marked by elongation and reorientation (“organization”) of Prox1^hi^ endothelial cells by postnatal day 0. The expression of the transcription factors Foxc2 and Nfatc1 and the gap junction proteins Gjc2, Gja1, and Gja4 were temporospatially regulated during this process. Foxc2 and Nfatc1 were coexpressed at P0, and combined Foxc2 deletion with calcineurin-Nfat inhibition disrupted early Prox1^hi^ endothelial organization, suggesting cooperative Foxc2–Nfatc1 patterning of these events. Genetic deletion of *Gjc2*, *Gja4*, or *Gja1* also disrupted early VV Prox1^hi^ endothelial organization at postnatal day 0, and this likely underlies the VV defects seen in patients with *GJC2* mutations. Knockout of *Gja4* or *Gjc2* resulted in reduced proliferation of Prox1^hi^ valve-forming cells. At later stages of blood flow, Foxc2 and calcineurin-Nfat signaling are each required for growth of the valve leaflets, whereas Foxc2 is not required for VV maintenance.

## Abstract (precis)

Patients with mutations in *FOXC2* and *GJC2* have reduced venous valve number and leaflet length. Experiments in mice by Lyons et al. show that Foxc2-Calcineurin-Nfatc1, and *Gja4*, *Gjc2*, *Gja1* regulate valve-forming cell organization. Foxc2, Calcineurin-Nfatc1, and blood flow regulate leaflet growth/maturation.

## Introduction

Venous valves (VVs) are widely distributed throughout veins and venules and facilitate unidirectional blood flow back to the heart, which acts to reduce the peripheral venous blood pressure (Phillips et al., 2004; Bergan et al., 2006; Meissner et al., 2007). VV failure is a central feature of the venous reflux that is seen in up to 40% of adults. Reflux leads to chronic venous hypertension (particularly in the lower limbs), which can cause pain, edema, hyperpigmentation, skin damage, and chronic intractable ulceration (Bergan et al., 2006; Meissner et al., 2007). Our understanding of the molecular mechanisms of VV development and subsequent maintenance is limited, and there are few therapeutic options to treat VV dysfunction (Bergan et al., 2006; Bazigou et al., 2011; Caggiati and Caggiati, 2013; Munger et al., 2013, 2016; Maleti et al., 2015). Elucidation of these mechanisms and understanding of how their dysfunction may lead to VV failure could facilitate the development of novel therapies to treat this condition.

Clinical studies have suggested a link between venous reflux and primary lymphedema, but its cause (i.e., a direct VV defect or an indirect effect, such as vein dilatation) has not been elucidated (Rosbotham et al., 2000; Mellor et al., 2007; Ostergaard et al., 2011). We have previously shown how genes (*Itga9*, *Efnb2*, *Fn-EIIIA*) regulating lymphangiogenesis also control VV formation and maintenance (Bazigou et al., 2011). Several transcription factors (Prox1, Foxc2, Nfatc1) and gap junction proteins (connexin [Cx]37, Cx43, and Cx47) have been implicated in the development of lymphatic valves (LV), cardiac valves, or VVs (de la Pompa et al., 1998; Winnier et al., 1999; Chang et al., 2004; Petrova et al., 2004; Norrmén et al., 2009; Kanady et al., 2011; Srinivasan and Oliver, 2011; Munger et al., 2013, 2016; Molica et al., 2014). Mutations in the genes encoding FOXC2, (*FOXC2*) CX47 (*GJC2*), and CX43 (*GJA1*) cause primary lymphedema in man (Fang et al., 2000; Ferrell et al., 2010; Ostergaard et al., 2011; Brice et al., 2013). *Gjc2^−/−^* and *Gja4^−/−^* (Cx37) mice have VV defects during VV maturation, but the timing of onset of those abnormalities during VV formation and the developmental processes underlying absent VVs have not, to our knowledge, been studied (Munger et al., 2013, 2016). Expression of Foxc2 and Nfatc1 is segregated to opposite leaflet surfaces during valve maturation, but their expression during VV initiation and potential cooperative signaling (as is seen in lymphatic endothelia) has not been studied (Norrmén et al., 2009; Munger et al., 2016). Similarly, during leaflet maturation Cx37 and Nfatc1 are not coexpressed in the same valve leaflet endothelial cells (ECs), but expression patterns in VV initiation remain undetermined (Munger et al., 2016).

In this study, we quantified the VV defects in the limbs of patients with primary lymphedema caused by mutations in *GJC2* or *FOXC2* and examined the earliest underlying mechanisms of VV failure caused by loss of those genes in mice. We identified reduced numbers of VVs and shorter VV leaflets in patients with lymphedema caused by mutations in *FOXC2* or *GJC2*. In mice, we characterized the induction and organization of valve-forming ECs (VFCs), which occur at an earlier point than previously identified, and show that they occur within highly spatially and temporally regulated domains of Prox1, Foxc2, Nfatc1, Cx37, Cx43, and Cx47 expression (Bazigou et al., 2011; Munger et al., 2016). Combined *Foxc2* deletion and inhibition of Nfat signaling (but not *Foxc2* deletion or Nfat inhibition alone) resulted in the failure of early VFC organization. Loss of connexin-encoding genes *Gja1*, *Gjc2*, or *Gja4* similarly resulted in a failure of VFC organization, and for *Gjc2* and *Gja4*, that resulted in reduced VFC proliferation. Both Nfat and Foxc2 signaling and blood flow were required for VV maturation to postnatal day 6 (P6). Unlike its role in LVs, we show that Foxc2 is not required in VV ECs for valve maintenance (Sabine et al., 2015).

## Results

### *FOXC2*, *GJC2*, and *GJA1* mutations in human VV disease

**Figure 1.**
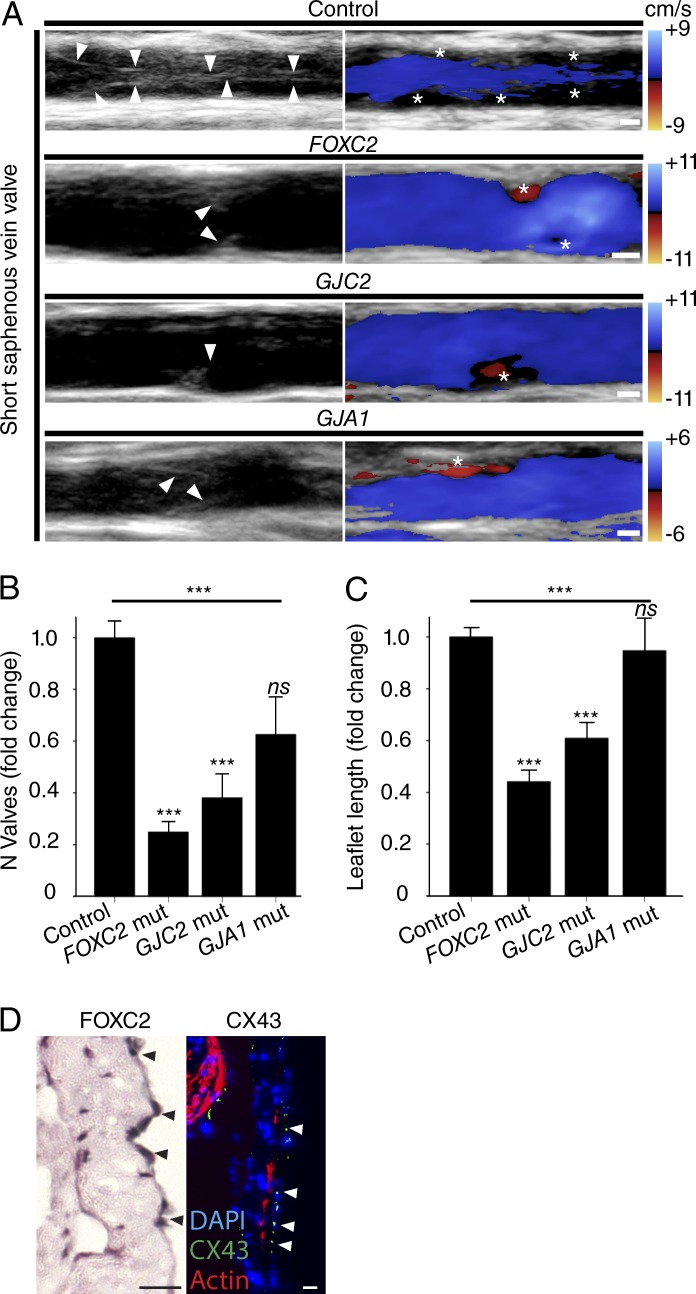
**Human VV phenotypes.** (A) VVs (arrowheads) were visualized in controls and in patients carrying mutations in the indicated genes, without (left) and with (right) power Doppler imaging of blood flow during a calf squeeze. An asterisk (*) marks stagnant/reversed flow in a VV sinus. Blood flow is from left to right. Bars, 1 mm. (B and C) Number (B) and length (C) of VV identified in patients carrying mutations in *FOXC2* (*n* = 8 patients), *GJC2* (*n* = 3), GJA1 (*n* = 1), and healthy controls (*n* = 10). See Tables S1, S2, and S3. ***, P < 0.0005 (ANOVA with Bonferroni correction). Error bars represent means ± SEM. (D) Immunolocalization (arrowheads) of FOXC2 and CX43 in adult human VV leaflets. Bar, 20 µm. Mut, mutation.

Understanding the relationship between primary lymphedema and VV dysfunction has been hampered by our inability to noninvasively quantify the presence and morphology of VVs in patients. Although VVs have been seen by ultrasonography, they have not been quantified on a systemic basis (Lane et al., 2007; Lurie and Kistner, 2012). We used conventional ultrasonography to visualize VVs in humans (Fig. 1 A). Quantification was reproducible for the number of VVs/vein in repeated scanning by different operators (*n* = 28 veins, intraclass correlation coefficient = 0.90, 95% confidence interval [CI] 0.63–0.91, P < 0.0005) and for leaflet length in repeated measurement by different operators (*n* = 15 VV, intraclass correlation coefficient = 0.94, 95% CI 0.83–0.98, P < 0.0005). This allowed quantification of the number of VVs and VV leaflet length in the upper and lower limbs (Tables S1 and S2). To limit scan duration, we scanned only the short saphenous, popliteal, basilic, and brachial veins. Consistent with postmortem studies, different numbers of VVs (Tables S2 and S3) were detected in control veins (means ± SD, popliteal 1.1 ± 0.7; short saphenous 3.4 ± 1.5; brachial 1.8 ± 1.2; basilic 2.4 ± 0.9, P < 0.0005, ANOVA) and, as expected, with different leaflet lengths (popliteal 6.8 ± 1.9 mm; short saphenous 4.0 ± 1.4 mm; brachial 3.9 ± 1.6 mm; basilic 4.9 ± 2.2 mm; P < 0.0005, ANOVA; Czarniawska-Grzesińska and Bruska, 2002; Iimura et al., 2003; Schweighofer et al., 2010; Moore et al., 2011). Subsequent comparisons were therefore analyzed as the fold change relative to the respective vein in the controls.

We found significantly reduced numbers of VVs in patients with mutations in both *FOXC2* (fold change ± SD 0.25 ± 0.32, P < 0.0005) and *GJC2* (0.38 ± 0.46, P < 0.0005; Fig. 1 and Tables 1 and 2) compared with age- and sex-matched controls. Those VVs that were present in the patients with *FOXC2* or *GJC2* mutations had shorter leaflets (0.44 ± 0.25, P < 0.0005 and 0.61 ± 0.28, P < 0.0005, respectively). VV defects were present in the veins of both upper and lower limbs in patients for both *FOXC2* and *GJC2* (not depicted). There also appeared to be fewer VVs in the single patient identified with lymphedema caused by a mutation in *GJA1* (CX43). Consistent with these findings, and in agreement with their expression pattern in mouse VVs, FOXC2, and CX43 were localized on adult human VVs, suggesting that human VV phenotypes may result from tissue-autonomous effects of the patients’ mutations (Fig. 1 D). CX43 is predominantly located on the lumen-facing leaflet surface, whereas FOXC2 is predominantly located on the sinus surface. We were unable to obtain typical, punctate CX47 staining using commercially available antibodies in human VVs (May et al., 2013). It is possible that the Cx47 expressed in VVs during development and early life is subsequently down-regulated in the adult human VV, as has been found in mice for VEGFR3 (Bazigou et al., 2011).

**Table 1.** Mean number of valves in controls and patients

Group	*n* VV/vein (normalized to control veins)		
	*n* veins analyzed	Mean *n* VVs/vein	SD
Control	68	1.00	0.53
*FOXC2*	64	0.25^a^	0.32
*GJC2*	24	0.38^a^	0.46
*GJA1*	8	0.63	0.41

Not all veins were scanned in all controls/patients.

a P < 0.0005 for *FOXC2* and *GJC2*, for mean *n* valves (ANOVA with Bonferroni correction).

**Table 2.** Mean VV leaflet length in controls and patients

Group	Leaflet lengths (normalized to control veins)		
	*n* VVs measured	Mean leaflet length	SD
Control	118	1.00	0.38
*FOXC2*	32	0.44^a^	0.25
*GJC2*	21	0.61^a^	0.28
*GJA1*	10	0.95	0.40

a P < 0.0005 for *FOXC2* and *GJC2*, for mean leaflet length (ANOVA with Bonferroni correction).

### Imaging early VV development in mice

To investigate possible underlying mechanisms for these human phenotypes, we next analyzed VV development in mice and focused on the proximal femoral vein (FV) because of the critical role of proximal deep VV in human disease and because a VV most consistently develops at this position in the mouse, allowing reliable identification of the earliest stages in its development (Meissner et al., 2007; Bazigou et al., 2011; Eberhardt and Raffetto, 2014). We had previously analyzed *opened* veins (by electron and confocal microscopy), which damages VV structures and precluded analysis of the anterior vein wall and complete staging of VV development (Bazigou et al., 2011). We therefore used whole-mount confocal microscopy of *unopened* veins to visualize the proximal FV before and during VV formation.

**Figure 2.**
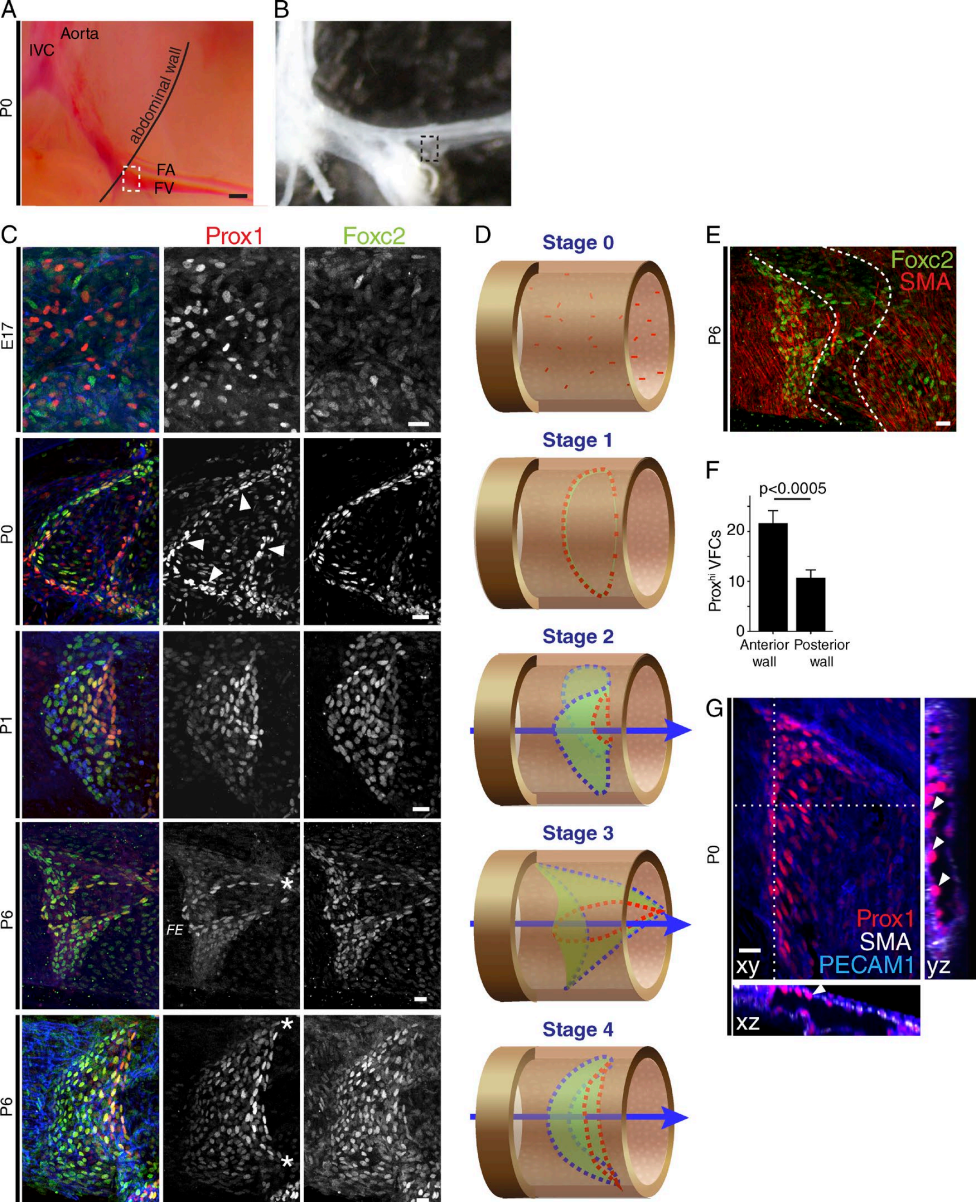
**VV development in the proximal FV.** (A and B) Micrographs before (A) and after (B) dissection to perform whole-mount imaging of the proximal FV valve (boxed region). IVC, inferior vena cava; FA, femoral artery. Bar, 200 µm. (C) Localization of Prox1 (red), Foxc2 (green), and PECAM1 (blue) at the indicated time points. An asterisk (*) marks single commissure at stage 3, and two commissures at stage 4. Arrowheads, reoriented and elongated VFCs. FE, free edge. Bars, 20 µm. *n* ≥ 6 VV/stage. (D) Corresponding schematics of stages in VV development: Prox1^hi^ free-edge cells (red), leaflets (green), and leaflet attachment to the vein wall (blue). Arrows, blood flow direction. (E) A peri-VV reduction (between dotted lines) in SMA-expressing cells (red). Bar, 20 µm. *n* > 6. (F) Quantification of elongated Prox1^hi^ valve cells on the anterior and posterior vein wall at P0. *n* = 9 (paired *t* test). (G) Arrowheads, anterior wall VFCs. Bar, 20 µm. Blood flow left to right in C–G.

Analysis from E17 to P6 revealed that initiation of VV formation occurs earlier than previously identified by imaging of opened veins or frozen sections (Fig. 2, A–C; Bazigou et al., 2011; Munger et al., 2016). At E17, Prox1 was initially widely expressed throughout the FV (not depicted), with increased expression in a heterogeneous subset of ECs, termed “Prox1^hi^” cells (as in LV; Sabine et al., 2012; Sweet et al., 2015). These cells were distributed across the vein in the region of prospective VV formation (Fig. 2, C and D; stage 0 of development). At P0, Prox1^hi^ nuclei had undergone elongation and reorientation and had organized to form a ring of VFCs around the vein (stage 1). Organization of VFCs is more prominent on the anterior wall of the FV, before full circularization (Fig. 2, F and G). This ring structure is the first time when the contiguous ring of spindle-shaped cells at the free edge of the valve leaflets (which is present throughout development and in adults) becomes identifiable. From that ring, a roughly circular leaflet develops toward the center of the FV, forming a marked constriction (stage 2). Part of the free-edge ring extends to the vessel wall to form the first commissure (stage 3). The free edge opposite that first commissure is not in contact with the vessel wall (see also Fig. S1 A; BALB/c mice). This is followed by formation of a second commissure (stage 4). At earlier stages, smooth muscle cells uniformly coat the FV but subsequently become sparser in the region of the VV sinus (Fig. 2 E at P6).

### Foxc2 and Nfatc1 cooperate to pattern initial organization

**Figure 3.**
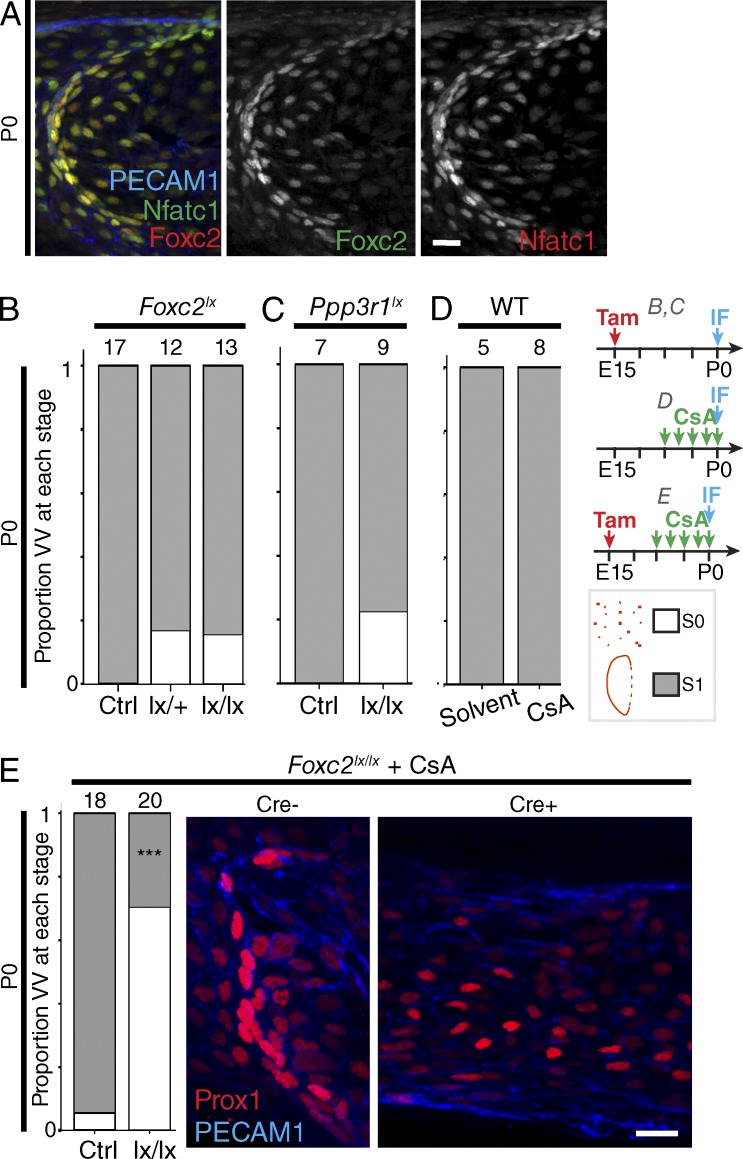
**Regulation of initial VV organization by Foxc2 and Nfatc1.** (A) Colocalization of Foxc2 (red) and Nfatc1 (green) in VFCs at P0. Foxc2 and Nfatc1 localization are also shown separately. *n* = 4. Bar, 20 µm. (B–E) The proportion of VVs identified at stage 0 (white) and stage 1 (gray) at P0 after deletion of *Foxc2* (B) or *Ppp3r1* (CnB1; C) from E15, or treatment with cyclosporin from E17 (D), or with *combined Foxc2* deletion and cyclosporin treatment (E). The number of VVs analyzed for each condition is indicated above each bar. ***, P < 0.0005, χ^2^. (E) Localization of Prox1 (red) is shown at P0 after combined *Foxc2* deletion and cyclosporin treatment. Bar, 20 µm.

The mechanism or mechanisms by which mutations in *FOXC2* and *GJC2* lead to defective VVs are unknown (Petrova et al., 2004; Mellor et al., 2007). We first analyzed the regulation of the initial ring of VFCs (stage 1), which forms by P0. *Foxc2*^−/−^ mice do not survive to birth, so we investigated the effect of conditional deletion of *Foxc2* using tamoxifen-inducible Cre recombinase under the control of the *Prox1* promoter (*Prox1;CreER^T2^*) in combination with floxed target alleles (Bazigou et al., 2011). Induction of Cre activity at E15 induced deletion in VV cells. However, neither heterozygous (*Foxc2^lx^*^/+^) or homozygous (*Foxc2^lx/lx^)* deletion produced a significant phenotype at P0 (Fig. 3 B). We therefore analyzed expression of the transcription factor Nfatc1, which is coexpressed with Foxc2 and is known to signal cooperatively during lymphatic vessel maturation (Norrmén et al., 2009). At P0, Nfatc1 was colocalized with Foxc2 in Prox1^hi^ VFCs and predominantly downstream of the forming valve (Fig. 3 A). Blocking Nfat signaling by homozygous deletion of the regulatory subunit of calcineurin (CnB1, encoded by *Ppp3r1*) in venous endothelium from E15, or administration of cyclosporin A (CsA; a well-characterized pharmacological inhibitor of calcineurin-Nfat signaling) from E17 resulted in the expected cytoplasmic localization of Nfatc1 (Fig. S2, A–C) but had no effect on VFC organization at P0 (Fig. 3, C and D; Chang et al., 2004; Norrmén et al., 2009; Sabine et al., 2012). To simultaneously inhibit signaling through both transcription factors, we treated *Prox1CreER^T2^;Foxc2^lx/lx^* mice with CsA. Combined *Foxc2^lx/lx^* deletion and Nfat inhibition resulted in significantly abnormal VFC organization (Fig. 3 E). Thus, *unlike* LVs, only combined inactivation of Foxc2 and calcineurin/Nfat signaling was able to disrupt normal VV organization (Norrmén et al., 2009).

### Connexins pattern VV organization at P0

**Figure 4.**
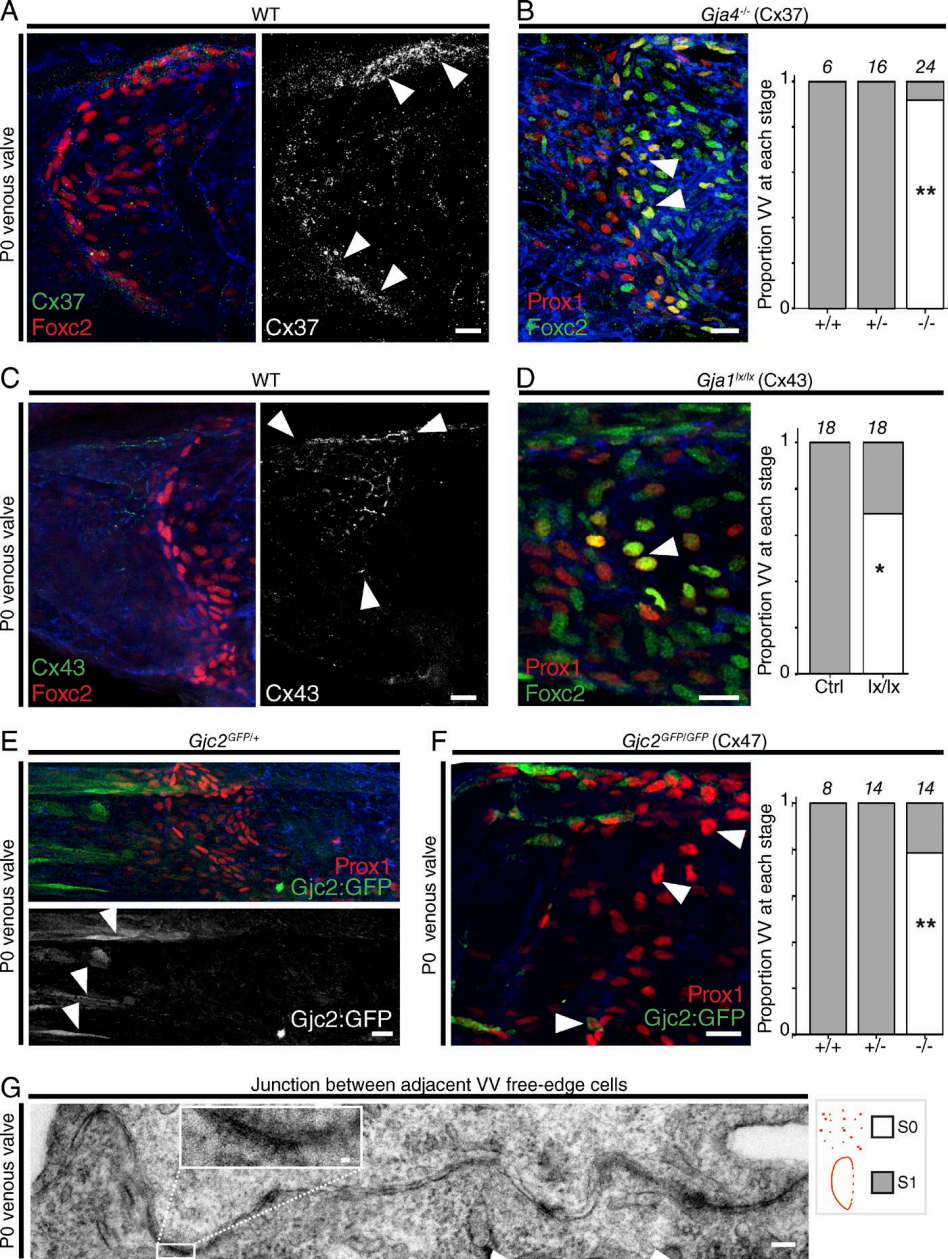
**Connexins pattern valve organization at P0.** (A–F) Expression in WT mice (A, C, and E) and the KO or conditional deletion loss-of-function phenotypes at P0 for *Gja4*, *Gja1*, and *Gjc2* (B, D, and F). (A) Immunolocalization of Cx37 (arrowheads), and (B) of Prox1^hi^ VFCs (arrowheads) in *Gja4^−/−^* mice. Immunolocalization of Cx43 (arrowheads; C), and of Prox1^hi^ VFCs (arrowhead; D) in *Prox1CreER^T2^;Gja1^lx/lx^* mice after induction of deletion at E15. (E and F) Immunolocalization of GFP (green, arrowheads in E) in *Gjc2^GFP/+^* reporter mice (E), and of Prox1^hi^ VFCs (arrowheads in F) in *Gjc2^GFP/GFP^* KO mice (F). *n* ≥ 6 for all images. Bars, 20 µm. Blood flow is from left to right throughout. Graphs in B, D, and F show the proportion of VVs identified at stage 0 (white) and stage 1 (gray) at P0 for the indicated genotypes, and number of VVs analyzed for each condition is given above each bar. *, P < 0.05; **, P < 0.005; χ^2^/Fisher’s exact test. (G) A tiled TEM image of the junction between two adjacent, free-edge cells at P0, shown at lower magnification in Fig. S3 K. *n* = 3. Bars: 100 nm; 10 nm in inset.

We next analyzed the expression of Cx37, Cx43, and Cx47 at the earliest stages of VV development in the FV (Fig. 4, A, C, and E). At E18 Cx37 was immunolocalized to lymphatics (not depicted) but was *not* detectable in the region of the FV where a VV subsequently always develops (Fig. S3 A). At P0 Cx37 was localized to VFCs, particularly in the upper and lower region of the vessel (Fig. 4 A). In *Gja4* (Cx37) homozygous constitutive KO mice, initiation of VV formation at the usual location and the typical boundary between higher distal Prox1 immunostaining and higher proximal Foxc2 immunostaining was preserved. VFCs were identified distributed across the full width of the vessel but appeared highly disorganized, failing to reach stage 1, whereas all WT and heterozygous KO littermate VVs reached stage 1, as expected (Fig. 4 B). We next analyzed the junctions between VFCs by transmission electron microscopy (TEM) at P0 and, despite the rearrangements that these cells were undergoing, confirmed the appearance of gap junctions (inset in Fig. 4 G and Fig. S3, J and K). Although gap junction–mediated, intercellular communication has been implicated in the regulation of Nfatc1 signaling in LV development in vitro, Nfatc1 immunolocalization remained nuclear in *Gja4^−/−^* mice, demonstrating that Cx37 is not critically required upstream of Nfat nuclear translocation in VV, albeit Nfatc1^hi^ nuclei were disorganized (as seen with Prox1 immunolocalization; Fig. S3 D; Kumai et al., 2000; Sabine et al., 2012).

In contrast to Cx37, expression of Cx43 was not detectable on most VFCs but was instead localized to ECs upstream of those cells, with the strongest Cx43 immunostaining in the superior half of the upstream vessel (Fig. 4 C). It is possible that junctions consisting of Cx43 allow communication between this upstream region and VFCs in some areas. The source of Cx43 at junctions between these cells (either VFC or upstream endothelia) remains unclear. Homozygous conditional deletion of *Gja1* (Cx43) resulted in the failure of organization of the VFCs downstream of that region, and VVs failed to reach stage 1, whereas WT littermates developed normally (Fig. 4 D).

Cx47 expression was studied using a *Gjc2^GFP^* (knock-in) reporter mice, with signal amplification using antibodies raised against GFP. Although no GFP signal was detected in the FVs of WT mice, expression was detected in cells dispersed throughout the FV in *Gjc2^GFP/+^* mice (Fig. 4 E and Fig. S3, G and H) and *Gjc2^GFP/GFP^* mice (Fig. S3 I). Compared with WT littermates, Prox1^hi^ VFCs showed a pattern of disorganization similar to loss of *Gja4* and *Gja1* at P0, but we could find very few VFCs expressing detectable Cx47 (GFP; Fig. 4 F), suggesting a non–VFC-autonomous role of *Gjc2* in modulating VFC organization.

**Figure 5.**
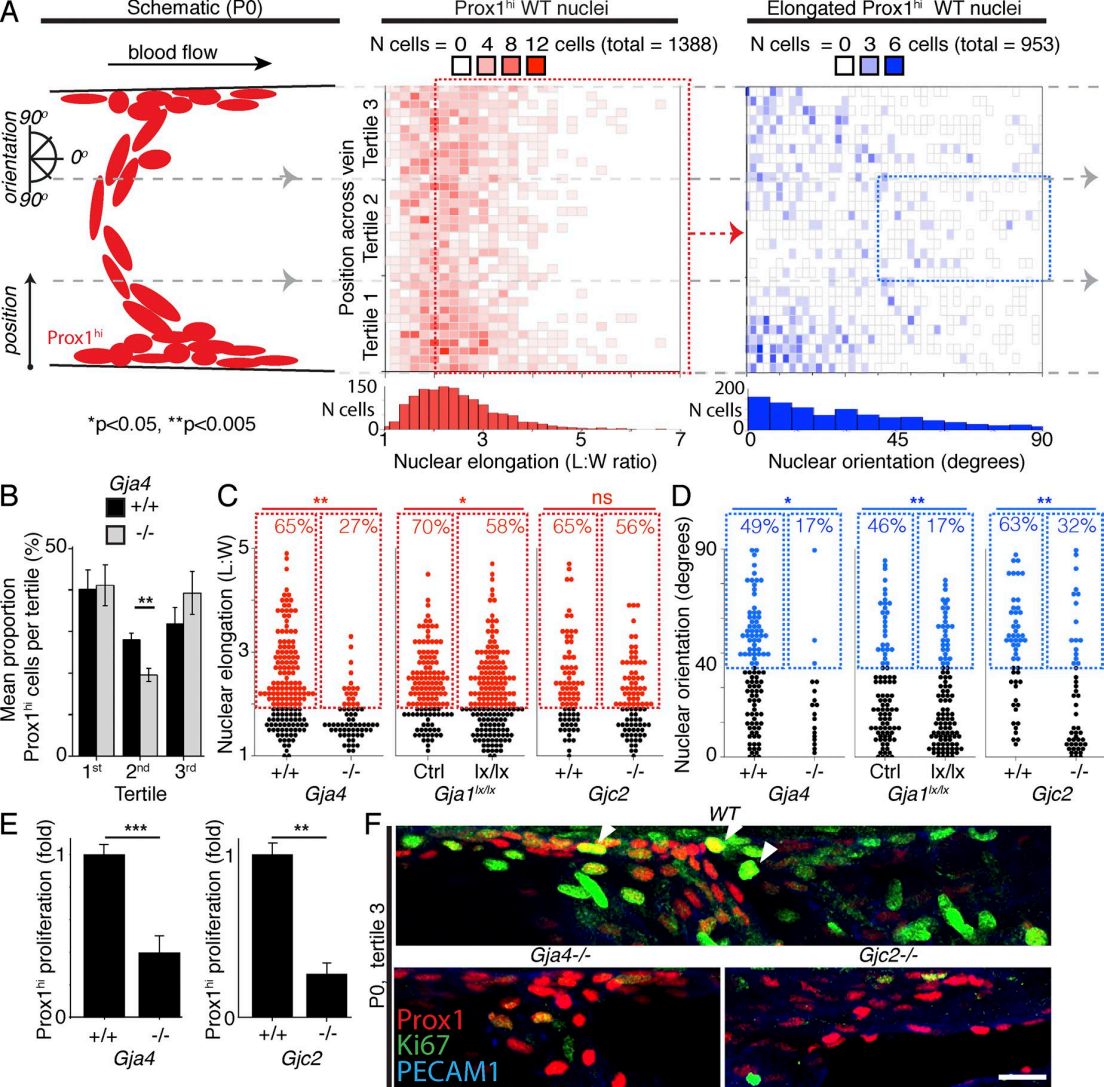
**Quantification of connexin phenotypes.** (A, left) The schematic indicates how position and orientation of Prox1^hi^ VFCs were measured in the middle and right panels. (Middle) Binned scatterplot of VFC elongation (length/width ratio) across the vein from superior (tertile 3) to inferior (tertile 1). *n* = 1,388 cells; 17 VVs. The red dotted box indicates elongated nuclei (length/width ratio ≥2), which were further analyzed in the right panel. (Right) Binned scatterplot of elongated VFC nuclear reorientation across the vein. The blue dotted box indicates nuclei rotated ≥40°. *n* = 953 cells; 17 VVs. (B) The proportion of Prox1^hi^ VFCs in the middle tertile is shown for the indicated genotypes.**, P < 0.005; *t* test. (C) The elongation of Prox1^hi^ nuclei from the central tertile of valves of the indicated genotypes is shown. Elongated nuclei (length/width ratio ≥2) are highlighted in red. (D) The reorientation of elongated Prox1^hi^ nuclei from the central tertile of valves of the indicated genotypes is shown. Reoriented nuclei (≥40°) are highlighted in blue. *, P < 0.05; **, P < 0.005; χ^2^. (B–D) *n* = 6 WT versus 8 *Gja4^−/−^* VVs; 6 WT versus 10 *Gja1^lx/lx^* VVs; 5 WT versus 5 *Gjc2^−/−^* VVs. Data for all genotypes and vein regions provided in Fig. S4. (E) Graphs indicate the mean number (as fold over WT littermate VVs) of Prox1^hi^;Ki-67^+^ proliferating VFCs for the indicated genotypes. *n* = 5 WT versus 6 *Gja4^−/−^* VVs; and 6 WT versus 6 *Gjc2^−/−^* VVs. **, P < 0.005; ***, P < 0.0005; *t* test. (F) Representative images are shown of Prox1 (red) and Ki-67 (green) immunolocalization in the upper region of the valve in *Gja4^−/−^* and *Gjc2^−/−^* mice and a WT littermate. Arrowheads indicate Prox1- and Ki-67–coexpressing cells. Bar, 10 µm. Images are a 6-µm-thick *z*-projection of 0.5-µm optical sections.

To further quantify the phenotypes resulting from loss of Cx37, Cx43, or Cx47, we first used *Prox1CreER^T2^* crossed with the *Rosa26mTmG* reporter, in which membrane-targeted GFP is expressed in Cre-recombined cells, enabling us to show that nuclear morphology (orientation relative to the vessel and length/width ratio) can be used as a surrogate marker for cellular morphology in P0 VFCs (Fig. S4, A and B; Muzumdar et al., 2007). We quantified Prox1^hi^ nuclear elongation and reorientation as components of VV organization (Fig. 5 A). Elongation did not correlate with reorientation (Fig. S4 C), and we therefore analyzed these attributes separately. Elongated Prox1^hi^ nuclei were significantly more reoriented in the central third of the vessel than in the upper and lower thirds (Fig. 5 A, right; and Fig. S4 D). We analyzed the proportions of Prox1^hi^ nuclei in each region of the vein, where loss of Cx37 (but not Cx43 or Cx47) resulted in a reduced proportion of Prox1^hi^ nuclei in the center of the vessel (Fig. 5 B). To quantify organization, we analyzed elongation and reorientation of nuclei within the central tertile, as previously described (Tatin et al., 2013). Loss of Cx37 or Cx43 resulted in reduced nuclear elongation, whereas loss of Cx37, Cx43, or Cx47 resulted in reduced reorientation of the nuclei (Fig. 5; data for all genotypes and vessel regions analyzed are provided in Fig. S4 E). Together, these data show that Cx37, Cx43, and Cx47 are each critical for the cellular reorganization that occurs to reach stage 1 of VV development.

To determine whether connexin loss resulted in failed proliferation of VFCs, we analyzed the proportion of Prox1^hi^ VFCs colabeled with Ki-67. This revealed reductions in the proportion of proliferating VFCs in both *Gja4^−/−^* and *Gjc2^GFP/GFP^* mice (P < 0.0005 and P < 0.005, respectively; Fig. 5, E and F). We could find no evidence of apoptotic VFCs in either of these KO lines (not depicted).

### Maturation of leaflets and commissures

**Figure 6.**
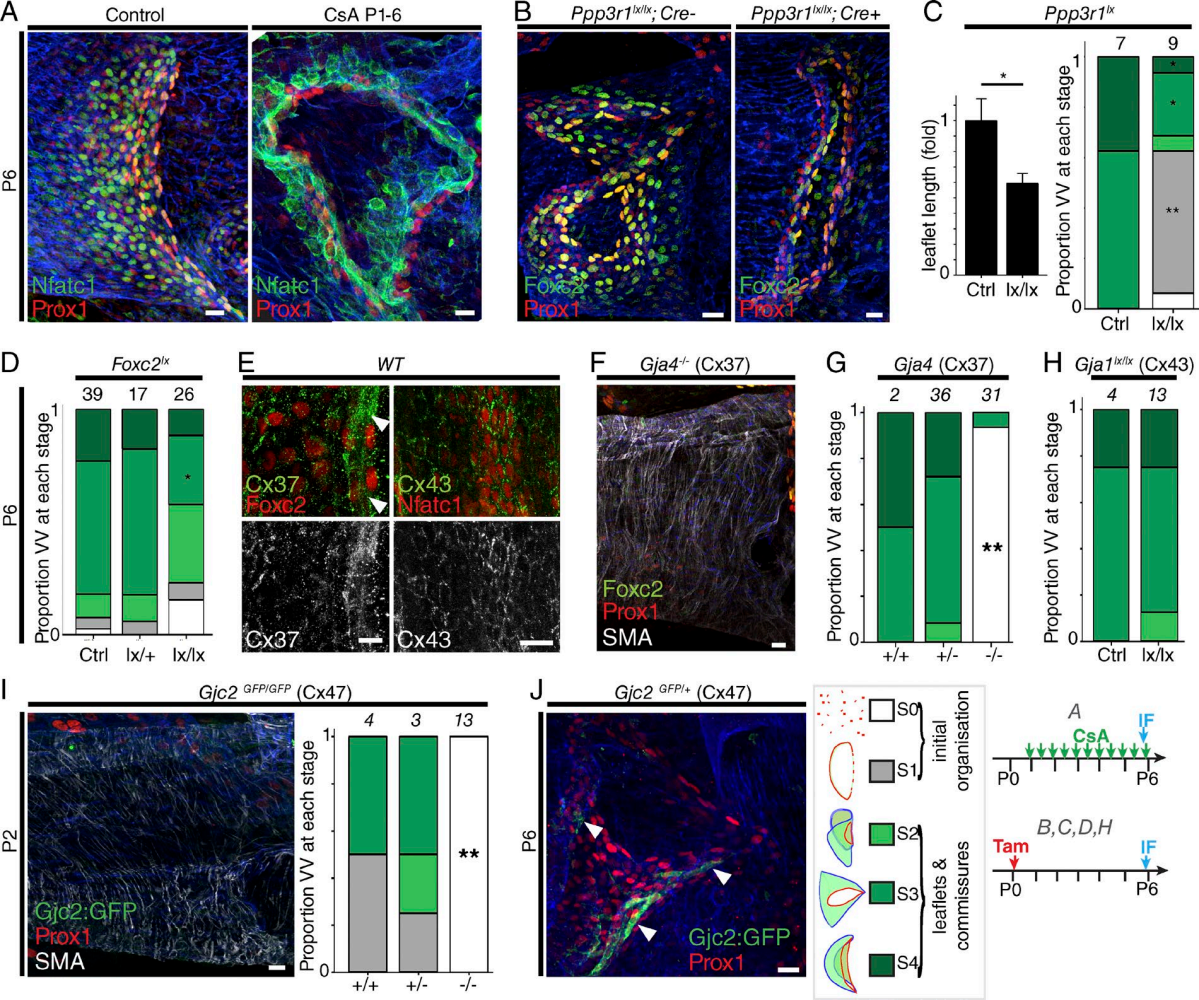
**Maturation of leaflets and commissures.** (A) Immunolocalization at P6 of Nfatc1 (green) and Prox1 (red) in VV of WT mice administered control solvent or cyclosporin (*n* = 5 versus 8 VVs, P < 0.05, χ^2^). (B) Immunolocalization of Foxc2 (green) and Prox1 (red) in VVs of mice of the indicated genotypes. *n* ≥ 6 per condition. (C and D) Quantification of leaflet length and stage of VV development in mice of the indicated genotypes. Error bars represent means ± SEM. *t* test for leaflet length. (C, D, G, H, and I) Colors represent developmental stage (see key). The number of VVs analyzed for each condition is given above each bar. *, P < 0.05; **, P < 0.005 (χ^2^ for developmental stage). (E) Immunolocalization of Cx37 (arrowheads) and Cx43 in VV leaflets at P6. *n* ≥ 4. (F) Localization of Prox1, Foxc2, and SMA at P6 in *Gja4^−/−^* vein. *n* ≥ 6 (G and H) Stages of VV development reached in mice of the indicated genotypes. (I) Localization of Prox1, GFP, and SMA in *Gjc2^GFP/GFP^* KO reporter vein at P2 (*n* = 4), and stages of VV development reached in mice of the indicated genotypes. (J) Localization of Cx47-expressing VV cells (arrowheads) of *Gjc2^GFP/+^* reporter mice at P6. *n* = 4. Multichannel images are reproduced in supplementary data. Bars, 20 µm. (A–J) Blue stain is PECAM1.

We next analyzed the maturation of VVs to P6, and asked whether Foxc2 or calcineurin-Nfat signaling is required for development of VV leaflets and commissures. In contrast to their cooperative roles in patterning the initial VV ring, calcineurin inhibition with CsA (Fig. 6 A), loss of CnB1 (*Ppp3r1^lx/lx^* deletion) at P0 (Fig. 6, B and C), or deletion of *Foxc2* alone (Fig. 6 D) were each sufficient to cause significant defects in leaflet/commissure development through to stages 3–4. There was no loss of the ring of rotated Prox1^hi^ free-edge cells, which remained intact even at P6 (Fig. 6, A and B), demonstrating that neither Nfatc1 signaling or Foxc2 are required for maintenance of this initial valve structure and the free-edge phenotype of these cells.

Cx37 remained expressed in VV leaflets at P6 (Fig. 6 E). Unlike the phenotype seen with loss of *Ppp3r1*, *Foxc2*, and to some extent, *Itga9* or *Efnb2* (Fig. S5), analysis of *Gja4^−/−^* VVs at P6 revealed complete absence of both leaflet structure and Prox1^hi^ or Foxc2^hi^ cells (Fig. 6 F), demonstrating that the early development of VV seen at P0 in *Gja4^−/−^* (Cx37) mice is subsequently lost, and Cx37 is required to maintain the free-edge cell phenotype. This was associated with failure to form the gap in smooth muscle actin (SMA)–expressing cells normally seen around the valve (Fig. 6 F). As expected, VV in *Gja4^+/−^* mice developed normally (Fig. S5 G). These results are consistent with previously reported findings in LVs and with the absence of VVs at P4 in *Gja4^−/−^* mice (Munger et al., 2013, 2016).

At P2, Cx43 was barely detectable in VV leaflets (Fig. S5 H), but at P6, it was clearly localized around Nfatc1-expressing ECs (Fig. 6 E). Conditional, homozygous *Gja1* deletion from P0 did not produce a significant phenotype at P6, suggesting that the critical requirement for Cx43 in VV development is restricted to events before or around P0 (Fig. S5 I).

GFP-expressing cells were almost entirely undetectable in *Gjc2^GFP/+^* VVs at P2 (Fig. S5 K), but by P6, Cx47-expressing cells were clearly detected within the valve (Fig. 6 J). In contrast, *Gjc2^GFP/GFP^* mice (lacking *Gjc2* expression) had no valve cells by P2, indicating loss of the Prox1^hi^ phenotype seen at P0. These results suggest that the critical requirement for Cx47 is restricted to events around P0 (Fig. 6 I and Fig. S5 L) and are in agreement with previous findings of absent VVs in femoral and other veins analyzed at later postnatal stages in *Gjc2^−/−^* mice (Munger et al., 2016). Notably, Cx47 is not required for the development of valves in all veins (e.g., the superficial caudal epigastric or proximal subclavian veins), consistent with our finding of ∼40% VVs remaining in peripheral veins of patients with mutated *GJC2* (Munger et al., 2016).

### A role for blood flow in VV development

**Figure 7.**
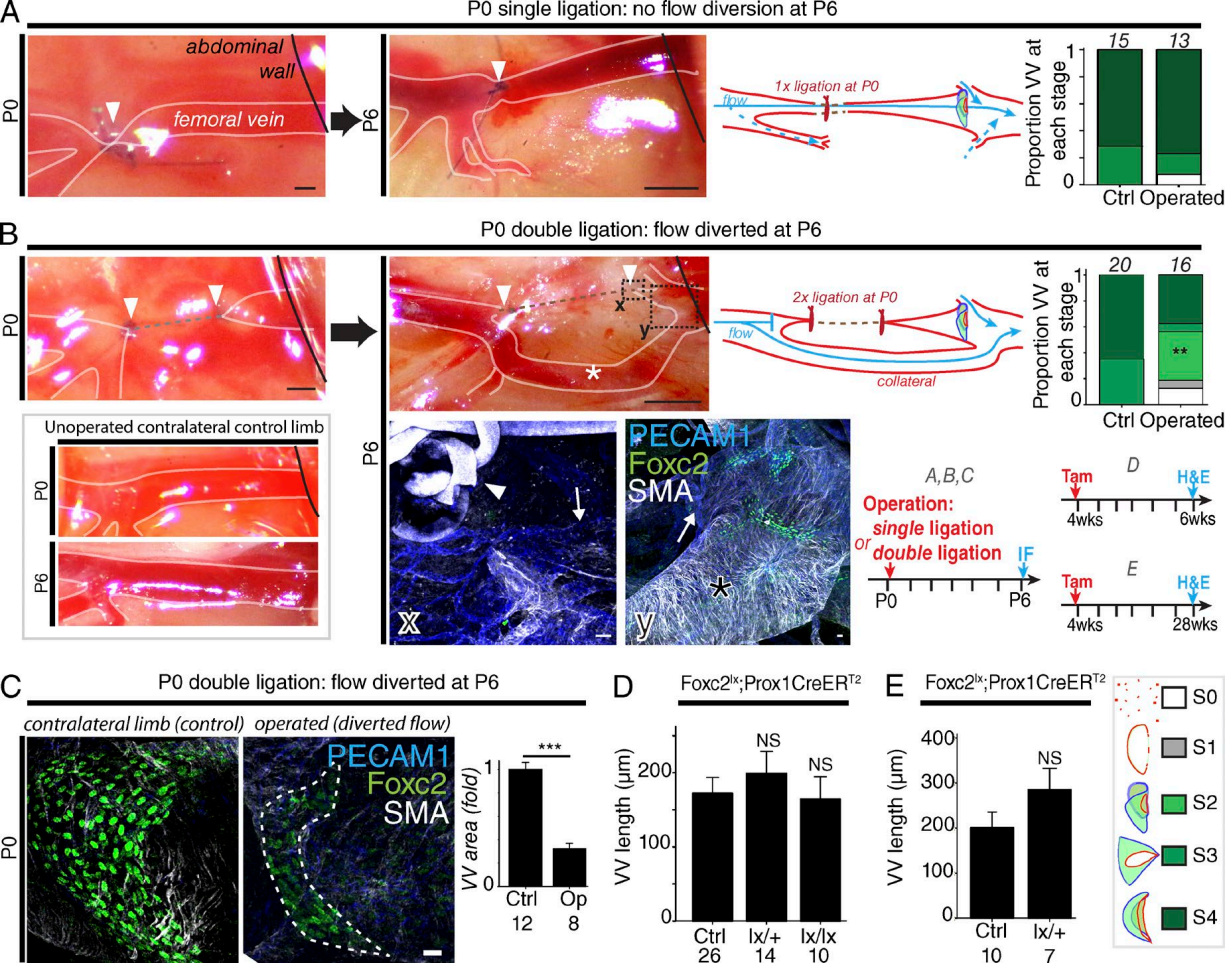
**Role of blood flow in valve growth and the regulation of VV maintenance.** (A and B) Representative images at P0 and P6 after single FV ligation (arrowheads in A) or double ligation and FV division (arrowheads in B) at P0, and quantification of the proportion of valves reaching each developmental stage at P6 in VVs of operated and unoperated limbs. The number of VVs analyzed is given above bars. An asterisk (*) indicates collateral vessel. Bars, 500 µm. **, P < 0.005, χ^2^. Dotted boxes X and Y indicate regions examined by immunofluorescence at P6. In X, arrowhead indicates suture, and the arrow shows the remodeling FV. In Y, the asterisk (*) indicates large collaterals, and the arrow shows the proximal FV. Bars, 20 µm in micrographs. (C) Localization of Foxc2 (green) and quantification of VV area in VV exposed to reduced flow (*n* = 8) and controls (*n* = 12). ***, P < 0.0005, *t* test. Bar, 20 µm. (D and E) Analysis of VV length (Hematoxylin-eosin) at 2 wk (D) and 24 wk (E) after *Foxc2* deletion. P = NS (ANOVA). The number of VVs analyzed is given below each bar.

Because several genes required for VV development, including *Foxc2* and *Gja4* (Cx37), are up-regulated by fluid shear stress, we hypothesized that blood flow may be required for normal VV leaflet growth (Sabine et al., 2012; Sweet et al., 2015). To alter blood flow across the developing VV, we ligated and divided the FV at P0, and analyzed the VV at P6. No thrombosis was seen, and pups operated on gained weight normally (P > 0.05 vs. unoperated littermates). We initially ligated the FV at P0 (Fig. 7 A), which did not result in visible diversion of blood flow (likely because of the small collaterals seen at the site of the ligation; not depicted), and valve development was unaffected. We then ligated the FV twice and divided the FV (Fig. 7 B), resulting in rerouting of blood via collaterals, with flow past the main valve region seen to reenter via those collaterals (Fig. 7 B). VVs exposed to those altered flow conditions showed reduced progression to the later stages of leaflet development compared with VVs exposed to unaltered flow conditions on the unoperated, contralateral side (Fig. 7 B) and were smaller (Fig. 7 C).

### Regulation of VV maintenance

Because patients with heterozygous *FOXC2* mutations showed severe VV defects, and *Foxc2* is required for LV maintenance, we next asked whether *Foxc2* was also needed for VV maintenance as well as development (Sabine et al., 2015). Induction of deletion in *Foxc2^lx/lx^* mice at 4 wk resulted in absent Foxc2 immunosignal 4 d later (not depicted), but interestingly, VV length at 6 wk (*Foxc2^lx/+^* or *Foxc2^lx/lx^*) or 28 wk (*Foxc2^lx/+^*) was similar to littermate controls (Fig. 7, D and E), demonstrating that EC Foxc2 expression is not required for VV maintenance at the timepoints analysed.

## Discussion

We have identified profound structural VV defects in patients carrying mutations in the genes encoding the transcription factor FOXC2 and the gap junction protein CX47. Crucially, we have extended our understanding of VV development in mice to the earliest endothelial events and have identified a temporospatially regulated pattern of transcription factor and gap junction protein expression around Prox1^hi^ VFCs. This is required for the organization of an initial ring of VFCs that is then critical for ongoing VV development. Using a genetic loss-of-function approach combined with drug inhibitors, we have demonstrated requirements for Cx37, Cx43, and Cx47 and provided evidence for Foxc2–Nfatc1 cooperative signaling in the regulation of VFC organization at P0. At later stages, we show requirements for Foxc2 and calcineurin–Nfat signaling and blood flow in regulating the maturation of valve leaflets.

### Human VV phenotypes

Valve defects were particularly severe in patients with *FOXC2* mutations, with 75% fewer valves, and remaining valves were almost half the length of valves in matched controls. This finding is consistent with the increased incidence of chronic venous insufficiency that we have previously identified in this group of patients (Brice et al., 2002; Mellor et al., 2007). Patients with mutations in *GJC2* had 62% fewer valves, and those present were substantially shorter than those seen in controls. Again, this is consistent with the studies of incompetent veins in those patients (Ostergaard et al., 2011). The human *GJC2* mutations studied here produce an aa substitution in the first extracellular loop (which may affect connexin docking) and one in the intracellular loop (Ostergaard et al., 2011; Molica et al., 2014). The mutation in *GJA1* (Cx43) results in a substitution (K206R) in the highly conserved SRPTEK motif of the second extracellular loop and may exert dominant negative effects on connexin docking, gap-junction function, and levels of WT protein (Brice et al., 2013; Molica et al., 2014). The finding of fewer VVs in a single patient (albeit without statistical significance) is consistent with the VV developmental defects seen in mice after *Gja1* deletion and requires exploration as further patients with *GJA1* mutations are identified.

### Transcriptional regulation of mouse VV formation

Our studies in mice extend previous descriptions of VV development, with our analysis of intact veins showing that initial events in VV formation occur earlier than we and others have previously identified (Bazigou et al., 2011; Munger et al., 2013, 2016). We also show that valve development is affected by the background of the mouse strain used (Fig. S1).

In lymphatic endothelium, Foxc2 and Nfatc1 are coexpressed, physically interact, and signal cooperatively to pattern vessel maturation (Norrmén et al., 2009). We examined proximal whole-mount vein samples and found that Foxc2 and Nfatc1 were coexpressed by VFCs and endothelium downstream of the organizing VFCs. We report that although neither endothelial Foxc2 or calcineurin–Nfat signaling alone is required for organization of initial valve-ring patterning, combined inhibition of Nfat signaling and *Foxc2* deletion results in complete disorganization of the valve-forming region. This leads us to suggest that there is a degree of redundancy in regulation at this point. It is difficult to fully exclude the possibility that a very low level of Foxc2 protein or calcineurin–Nfat signaling remained after *Foxc2/Ppp3r1* deletion and inhibition of Nfat signaling. However, we could find no Foxc2 immunosignal present in VVs 4 d after induction of deletion (unpublished data) in 4-wk-old mice, suggesting that Foxc2 depletion in VVs was efficient. In our experiments, we either deleted *Ppp3r1* (CnB1) or administered CsA. Although we did not observe specific staining in developing VVs using antibodies raised against Nfatc2, Nfatc3, or Nfatc4 (unpublished data), we cannot fully exclude possible roles for these proteins.

### Connexins pattern VFC organization

Although variation in connexin expression in veins at P0 has recently been reported, the expression was not related to positioning at the sites of VV formation, which was not described until P4, when VV leaflets are already maturing (Munger et al., 2016). Here, whole-mount techniques enabled us to demonstrate that expression of Cx37, Cx43, and Cx47 is specifically temporospatially regulated relative to VFCs at an earlier stage, between E18 and P0. Although Cx37 was expressed by Prox1^hi^ VFCs, Cx43 and Cx47 were predominantly expressed by cells upstream of the VFCs, rather than by VFCs themselves. Despite that, loss of *Gja1*, *Gjc2*, and *Gja4* all resulted in failure of organization of the early Prox1^hi^ VFCs, characterized by loss of Prox1^hi^ elongation and reorientation. Cx37 loss resulted in a lower proportion of VFCs in the central portion of the vein, but that was not seen for loss of Cx43 or Cx47. Loss of Cx37 or Cx47 resulted in reduced VFC proliferation. It remains unknown whether this is a direct effect of the loss of gap junctions or because of the failure of VFCs to organize normally, which could lead to reduced cell–cell signaling and loss of reinforcement of VFC identity. The loss of proliferation seen with *Gja4* KO is surprising because in other settings loss of Cx37 was associated with increased proliferation (Burt et al., 2008; Morel et al., 2010; Kanady et al., 2015). Although we did not detect apoptotic VFCs, we cannot exclude the possibility that any apoptotic cells were rapidly washed downstream. Collectively, our data suggest that in the setting of VVs it may be a failure of Prox1^hi^ cell organization (and resulting breakdown of inter-VFC communication) that results in the subsequent failure of further valve development in these mice.

Our TEM analysis of cell–cell junctions between free-edge (Prox1^hi^) VFCs at P0 shows the presence of gap junctions. It is possible that groups of ECs could develop discrete regions of gap-junction intercellular communication, for example, upstream of the initial Prox1^hi^ ring (Cx43, Cx47) or between free-edge cells themselves (Cx37), allowing for patterning of development by restricted regional endothelial signal propagation (Tallini et al., 2007; de Wit and Griffith, 2010; Pfenniger et al., 2012). Regional variations in endothelial connexin expression have important roles in cardiac valve formation, for example, patterning of the transcriptional activity of Nfatc1 by Cx45 in endocardial-cushion ECs (where deletion of Cx45 abrogated Nfatc1 signaling; Kumai et al., 2000). Sometimes connexins have roles entirely independent from intercellular communication. For example, Cx43 modulates cell polarity and directional cell migration (Francis et al., 2011). Although this cell-autonomous role could not entirely explain the disorganization of VFCs because we could find no Cx43 in most of those cells at P0, Cx43 loss could disrupt behavior of ECs immediately upstream of the Prox1^hi^ cells. The population(s) of cells contributing to VV have not been defined and could include contributions from that upstream region, for example, by becoming leaflet ECs or by communicating with valve cells to regulate their behavior. Interpretation of connexin phenotypes is further complicated by the formation of heteromeric and/or heterotypic channels (Goodenough and Paul, 2009; Molica et al., 2014).

### Patterning of valve position

Despite the Prox1^hi^ disorganization phenotype seen with loss of *Gja1*, *Gjc2*, and *Gja4*, VFCs were nonetheless located at the normal site of VV formation, and in a broad region across the vessel, suggesting that other factors are responsible for the positioning of the VV along the vein, the initial VFC specification, and for the overall patterning of that initial ring of VFCs. The finding of roles for *Gja1* and *Gja4* in patterning VFC reorientation and elongation at P0 is consistent with both the molecular similarity between VVs and LVs that we previously reported (Bazigou et al., 2011) and previous studies of absent VVs in *Gja4^−/−^* and *Gjc2^−/−^* mice identified at later ages (Munger et al., 2013, 2016).

### Regulation of leaflet maturation and maintenance

Our data indicate differential requirements for maintenance of the free-edge cells during later development beyond P0. To more accurately assess the VV phenotype seen with loss of *Itga9* and *Efnb2*, we performed whole-vein confocal imaging of mice with postnatal deletion of *Itga9* and *Efnb2* induced from P0 (Bazigou et al., 2011). As expected, at P6 a severe phenotype was seen in both mutants with complete loss of VV leaflets but with some residual Prox1^hi^ VFCs remaining (Fig. S5, A–C; Bazigou et al., 2011). By comparison, valve cells were completely absent at P2 with loss of Cx47, and at P6 with loss of Cx37. This phenotype was more severe than that identified with loss of integrinα9, ephrin-B2 (when many Prox1^hi^ cells remained) or CnB1 (when a strikingly complete VFC ring remained). This restricts the requirement for Nfatc1 to extension of the VV leaflets and not to maintenance of VFC identity. Our results suggest that Cx37 and Cx47 are required for the early stability of the VFC ring phenotype, as has previously been suggested for Cx37 in LVs (Sabine et al., 2012). These results are also in accordance with a study of failed invagination of lymphovenous valves in *Gja4^−/−^* mice (Geng et al., 2016). Cx37 remains highly expressed by VVs and may additionally be required for VV maintenance. In *Gjc2^GFP/+^* mice, Cx47-expressing cells were clearly identified at P6, suggesting that Cx47 could also have ongoing roles in maintenance. Delineation of possible roles for Cx37 and Cx47 after P0 will require a conditional loss-of-function approach. The role of Cx43 was examined by conditional deletion in ECs, and we found that connexin was required for early but not later stages of valve development. *Gja4^−/−^* and *Gjc2^GFP/GFP^* mice failed to develop an area of reduced SMA-expressing cells around the valve, most likely secondary to loss of the valve ECs (Bouvrée et al., 2012; Jurisic et al., 2012).

Calcineurin–Nfat signaling was critical for leaflet elongation and commissure formation, consistent with its requirements for leaflet growth in the aortic valve and LVs (Johnson et al., 2003; Chang et al., 2004; Sabine et al., 2012). Conditional homozygous deletion of *Foxc2* similarly produced a phenotype during leaflet maturation. In contrast to the requirement for *Foxc2* in LV maintenance (Sabine et al., 2015), we found that *Foxc2* was not important for VV maintenance during the time frame investigated. The timing of the onset of VV failure in patients with *FOXC2* mutations is unknown; further work should explore whether VVs in those patients develop normally and later regress or fail to develop at all. We speculate that other transcription factors (e.g., FOXC1) could regulate VVs and compensate for loss of FOXC2 (Fatima et al., 2016).

### The role of blood flow

The regulators of Cx37, Cx43, and Cx47 in VV formation remain unclear. In vitro and in vivo experiments indicate that Foxc2 regulates expression of Cx37 in lymphatic endothelium (Sabine et al., 2012; Munger et al., 2013). Although both are expressed by VFCs, the lack of VFC phenotype seen after *Foxc2* deletion suggests that other factors can compensate for the loss of Foxc2 in these cells at P0. (Munger et al., 2013; Kanady et al., 2015). For example, Klf2 regulates Cx37 expression in blood endothelium in regions exposed to high laminar shear stress (Pfenniger et al., 2012). Alternatively, induction of a GATA2/Foxc2/Prox1 pathway by oscillatory shear has been proposed as a mechanism for initiation of LV formation (Sabine et al., 2012; Kazenwadel et al., 2015; Sweet et al., 2015). GATA2 is expressed by venous VFCs (unpublished data), and a similar process may regulate VV initiation. The early protrusion of VFCs into the lumen and the associated shear exposure may provide a further mechanism to regulate expression in those cells. Our finding of smaller, less-developed VVs after reduction in blood flow through the FV (albeit during VV maturation, not initiation) is consistent with that concept. We cannot confirm, however, that the effect is valve specific rather than a general effect on EC growth.

### Conclusions

Patients with mutations in *FOXC2* and *GJC2* have globally reduced numbers of VVs and shorter VV leaflets. Foxc2 and calcineurin–Nfatc1 signaling cooperate to organize the initial ring of VV-forming cells. Cx37, Cx43, and Cx47 are critical for early organization of VFCs at P0, and failure of this process likely underlies abnormal VVs identified in patients with mutations in *GJC2*. Foxc2 expression in valve ECs is not required for VV maintenance.

## Materials and methods

All human and animal studies were performed in accordance with national regulations and ethical approvals (National Research Ethics Service Committee, South East Coast 10/H0701/68, 12/LO/1164, and the UK Home Office).

### VV ultrasonography

The following veins underwent ultrasonographic evaluation: the brachial (antecubital fossa to axillary vein; medial brachial vein, if paired), basilic (antecubital fossa to axillary), popliteal (adductor hiatus to trifurcation), and short saphenous (saphenopopliteal junction, if within 10 cm of the knee skin-crease, to 20 cm below the knee). Veins were visualized along their entire length, switching between longitudinal and transverse views to detect VVs, using a Philips Healthcare IU22 ultrasound machine with L17-5MHz/L9-3MHz probes. Participants with any history of deep vein thrombosis were excluded as were ablated or operated veins. Images and cine loops were recorded of each VV in B mode and with color Doppler. VV maximum leaflet measurements were obtained offline (Xcelera catheterization laboratory software; Philips Healthcare). For each vein, the number of VVs and VV length was normalized to the mean value in the respective control veins.

### Human genotyping

The screening of patient with *FOXC2* mutations was performed by the South West Thames Regional Genetics Service at St George’s Hospital, University of London. Screening for *GJC2* and *GJA1* mutations was performed as previously described (Ostergaard et al., 2011; Brice et al., 2013).

### Mouse lines

In brief, BALB/c, MF1, FVB, C57BL/6, and CD1 WT mice were obtained from Charles River UK. WT analyses were performed in BALB/c mice, unless indicated. *Prox1CreER^T2^* (Bazigou et al., 2011), *Foxc2^lx^* (Sasman et al., 2012), *Gja4^−/−^* (Simon et al., 1997), *Gjc2^GFP^* (Odermatt et al., 2003), *Gja1^lx^* (Calera et al., 2006), * Ppp3r1^lx^* (Zeng et al., 2001), and Rosa26^mTmG^ (Muzumdar et al., 2007) mice have been described previously and were maintained on C57BL/6 backgrounds, except for *Prox1CreER^T2^;Foxc2^lx^*, which was back-crossed onto BALB/c mice. For the induction of Cre activity in *Prox1CreER^T2^* mice, tamoxifen/4OH–tamoxifen (in sunflower oil; Sigma-Aldrich) was injected i.p. with either 5 mg at E15 for analysis at P0, or 50 µg at P0 for analysis at P6 (Bazigou et al., 2011). 37.5 µg/g mouse weight progesterone was given i.p. at E15 + E18, and embryos were analyzed at “E19” (equivalent to P0). In all experiments, we compared VVs in *Prox1CreER^T2^*^+^ with *Prox1CreER^T2^*^−^ littermate controls. For drug inhibition of Nfat nuclear translocation, 50 µg/g mouse weight CsA (EMD Millipore) was administered i.p. twice per day at P1–P6, and VVs were analyzed at P6. For combined deletion of *Foxc2* and calcineurin inhibition, deletion was performed from E15, and CsA at 50 µg/g mouse weight i.p. twice per day from E17 to E19. For induction of deletion at 4 wk, mice received 1 mg Tam i.p. once per day for 2 d. For flow alteration at P0, pups were anaesthetized (Univentor 4000) with isoflurane, and the femoral artery and vein (which are inseparable at P0) were ligated (10/0 vicryl), and the vein was divided before closure (10/0 vicryl); postoperative care was provided with the mother.

### Electron microscopy

For TEM, P0 pups were culled and perfused via the aorta with heparinized PBS (hPBS; 25 mg/liter; MP Biomedicals) before fixation overnight in glutaraldehyde (2.5% vol/vol in 0.1 M cacodylate buffer, pH 7.4, 4°C) and postfixation in osmium tetroxide (1% wt/vol in 0.1 M cacodylate, pH 7.4, 4°C) for 1.5 h. For visualization of mouse gap junctions, samples were immersed in 1% osmium tetroxide: 1.125% potassium ferrocyanide for 1 h, followed by en block staining with 1% aqueous uranyl acetate. All samples were dehydrated through graded ethanols, equilibrated with propylene oxide, infiltrated with epoxy resin (TAAB Laboratories Equipment), and polymerized at 70°C for 24 h. Semithin sections (0.5 µm) were cut and stained with 1% Toluidine Blue. Ultrathin sections (50–70 nm; Reichert-Jung ultramicrotome) were mounted and contrasted using uranyl acetate/lead citrate for examination (H7600, 80 kV, AMT digital camera; Hitachi).

For scanning electron microscopy (SEM), pups were culled and perfused via the aorta with hPBS before opening the femoral and iliac veins and overnight fixation at 4°C in glutaraldehyde (2.5% vol/vol in 0.1 M cacodylate buffer, pH 7.4) and postfixation in 1% (wt/vol) osmium tetroxide for 40 min at room temperature. After washing, samples were dehydrated through graded ethanols before critical point drying (Polaron E3000, Quorum Technologies) and mounting with conductive carbon cement (TAAB Laboratories Equipment) on aluminum pins (TAAB Laboratories Equipment) and gold sputter coating (Emitech K550X; Quorum Technologies) for examination (S3500N, 20 kV, high vacuum mode; Hitachi). VV leaflet length and position were measured in National Institutes of Health (NIH) ImageJ software.

### Immunohistochemistry

#### Human

For localization of connexins in human VVs (obtained from patients undergoing coronary artery or lower limb bypass grafting), 10-µm frozen sections were thawed and fixed in −20°C MeOH before permeabilization in 0.2% Triton X-100, charge neutralization in 0.5 M NH_4_Cl, blocking in 2% BSA, and incubation with primary antibody. For localization of FOXC2, after fixation in −20°C acetone and quenching of endogenous peroxidase using 3% H_2_O_2_, blocking (X0909; Dako), and incubation with primary antibody, signal was amplified using polymer-HRP (MP-XCP; Menarini Group), according to the manufacturer’s instructions. Localization of integrinα9 was performed on formalin-fixed, paraffin-embedded sections, using tyramide amplification (PerkinElmer), according to the manufacturer’s instructions.

#### Mouse

Mice were culled and perfusion fixed via the aorta and femoral vein with hPBS, followed by fixation with 4% formaldehyde, and then further fixed for 24 h. The external iliac and femoral veins were excised with surrounding muscles and processed for paraffin wax embedding and hematoxylin-eosin stains. 5-µm sections were photographed using a MicroPublisher 3.3 real-time viewing camera mounted on a Leitz DMRB microscope with PL Fluotar ×10, ×20, and ×40 lenses (Leica). Maximum leaflet length of each valve was measured in NIH ImageJ.

### Whole-mount immunostaining

Mice were culled and perfused with hPBS via the aorta before fixation in 4% paraformaldehyde, followed by blocking in 3% vol/vol donkey serum in 0.3% Triton X-100. Samples were further dissected before incubation with primary antibodies and were washed before localization with fluorophore-conjugated secondary antibodies. For colocalization of Nfatc1 and Foxc2, samples were blocked with Fab (donkey anti-goat IgG H + L, 100 µg/ml; Jackson ImmunoResearch Laboratories, Inc.) between sequential incubation with antibodies raised against Nfatc1 followed by Foxc2. Samples were then further dissected and mounted in Prolong Gold (Thermo Fisher Scientific). The consistent site of VV formation in unopened mouse FV was visualized by confocal microscopy (SP5; Leica Biosystems) to produce Z projections (ImageJ; NIH) of median filtered (Leica Application Suite Advanced Fluorescence/ImageJ, except for connexin localization) stacks. VV Prox1^hi^ nuclear elongation (length/width ratio) and reorientation (relative to the long axis of the vessel) were quantified in *z*-projections in ImageJ, as previously described (Tatin et al., 2013). Reorientation was analyzed in nuclei with length/width ratio ≥2. The position of each nucleus was taken as the center of the length measurement, and nuclear centile position across the vein was calculated (Excel; Microsoft) and used to allocate nuclei into tertiles according to position across the vein. For analysis of proliferating VFCs, Prox1^hi^;Ki-67^+^ nuclei were counted in alternate 1.5 µm optical sections (ImageJ; NIH) and presented as a proportion of total Prox1^hi^ VFCs. A reduced *z*-projection (4 slices, 6 µm) is shown (Fig. 5 F).

### Antibodies

#### Immunohistochemistry

Antibodies were raised in rabbits to Cx43 (3512; Cell Signaling Technology), sheep to FOXC2 (AF5044; R&D Systems), Foxc2 (AF6989; R&D Systems), and goat to Integrinα9 (BAF3827; R&D Systems).

#### Whole mount

Antibodies were raised in rabbit to Cx43 (3512; Cell Signaling Technology), Cx37 (CX37A11; Alpha Diagnostics International), Prox1 (11-002P; AngioBio), cleaved caspase 3 (9661; Cell Signaling Technology), Ki-67 (ab15580; Abcam), sheep to Foxc2 (AF6989; R&D Systems), goat to Nfatc1 (AF5640; R&D Systems), Prox1 (AF2727; R&D Systems), and GFP (ab6658; Abcam) rat to PECAM1 (BD clone MEC 13.3) and mouse to α-SMA (clone 1A4 conjugated to Cy3 [Sigma-Aldrich] or FITC [Abcam]).

#### Signal detection

Secondary antibodies or streptavidin were conjugated to Dylight-405/488/550/649 (Jackson ImmunoResearch Laboratories, Inc.) and streptavidin-HRP/alkaline phosphatase (Dako). IgG controls were nonimmune sheep/rabbit/goat IgG (R&D Systems).

### Statistical analysis

For human ultrasound, age and sex matching was tested, respectively, by ANOVA (with the Bonferroni correction) and Fisher’s exact test. All comparisons of human VV disease phenotypes were performed using ANOVA with Bonferroni correction. In mice, for VV developmental-stage 0–4 quantification, data represent the proportion of analyzed valves reaching each developmental stage. P-values represent differences in the proportion of valves at each stage versus WT littermates (χ^2^/Fisher’s exact test). For quantification of connexin-deletion phenotypes (Fig. 5), the proportion of nuclei elongated ≥2, and for elongated nuclei, reoriented ≥40°, was compared using χ^2^ tests, as previously described (Tatin et al., 2013).

For analysis of *CnB1*-deleted VV (Fig. 6), leaflet length was compared using a standard template in *z*-projections (independent samples *t* test). VV area was compared using an independent-samples *t* test. Abnormal VV development after CsA administration to P6 was compared by χ^2^ test. For VV maintenance, an independent-sample *t* test was used for two groups, and ANOVA was used for three groups. P < 0.05 was considered significant. All analyses were performed using IBM SPSS software (version 22).

### Online supplemental materials

Further details including patient characteristics, VV variability in mouse strains, and individual channels for multi-channel images are provided in online supplemental materials. Fig. S1 shows the variability in valves in different strains of mice. Figs. S2 and S3 show the regulation of initial VV organization (Cn–Nfat signaling). Fig. S4 shows the quantification of connexin phenotypes. Fig. S5 shows the maturation of leaflets and commissures. Table S1 shows the characteristics of study participants, and Table S2 shows the number of valves per vein in the control group, whereas Table S3 provides leaflet lengths per vein in the control group.

## Supplementary Material

Supplemental Materials (PDF)
