# Supplementary material for: Human venous valve disease caused by mutations in FOXC2 and GJC2
Source: J Exp Med. 2017 Aug 7;214(8):2437–52. doi: 10.1084/jem.20160875 (PMC5551565; doi:10.1084/jem.20160875)
Supplement: Supplemental Materials (PDF) [file JEM_20160875_sm.pdf]

The Journal of Experimental Medicine

SUPPLEMENTAL MATERIAL

Lyons et al., https://doi.org/10.1084/jem.20160875

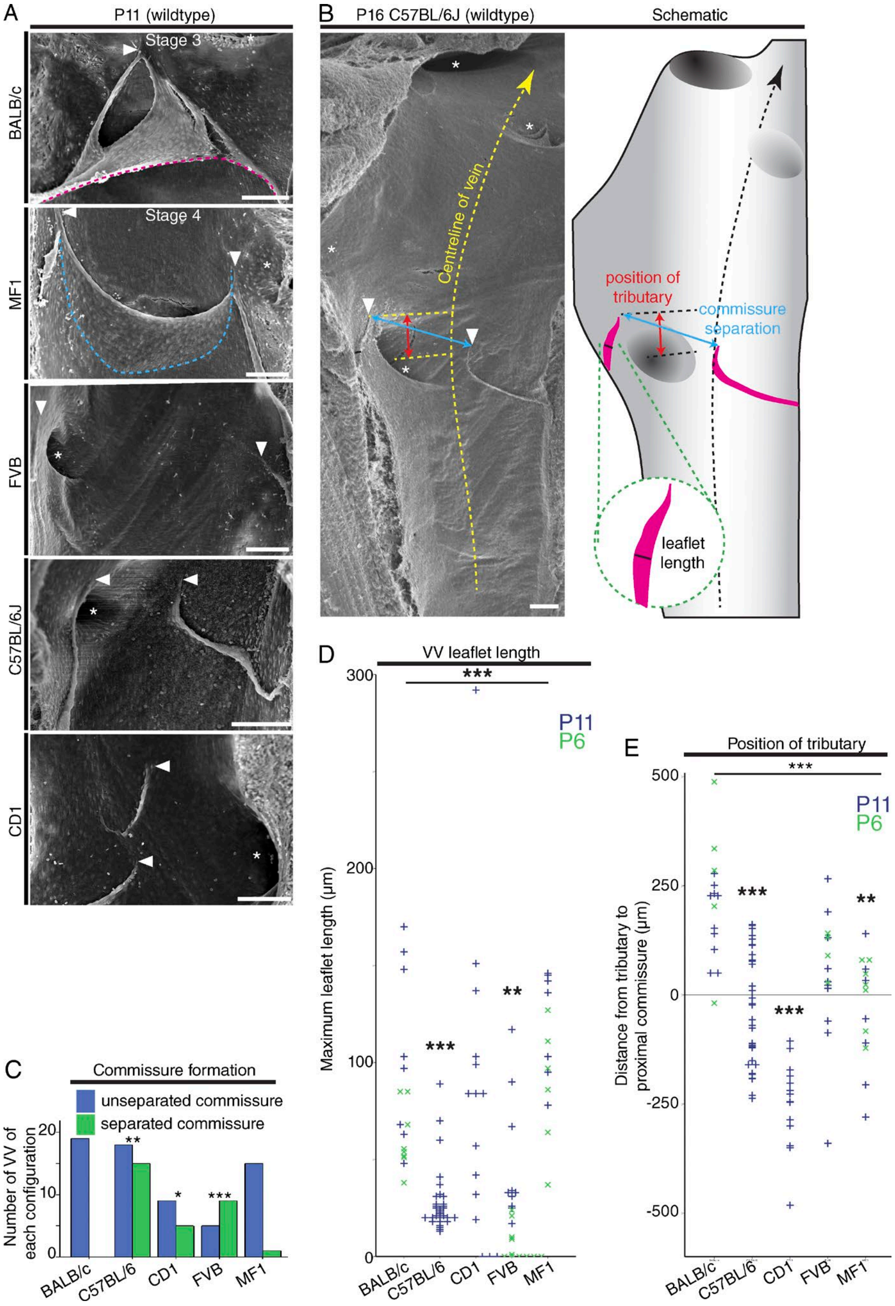

Figure S1. **Variability in valves in different strains of mice.** (A–E) We noted variant VVs in some WT C57BL6/J mice and compared VVs in several strains. (A) Representative images ($n \geq 6$ per condition, as indicated in C) of VVs in WT mice from the indicated strains at P11, indicating the variant of separated commissures around an unusually close tributary commonly seen in FVB (n = 14 VVs), C57BL6/J (n = 33), and CD1 (n = 14) strains, but rarely in MF1 (n = 16) and not in BALB/c (n = 19) mice. The pink dotted line indicates where leaflets have been cut during opening of the vein, and the blue dotted line marks the junction of the leaflet with the vein wall. Arrowheads indicate commissures (BALB/c, MF1) or abnormally separated commissures (FVB, C57BL6/J, CD1). The orifice of the tributary normally just downstream of the valve is marked with an asterisk (*), and lies between the separated commissures in images from FVB, C57BL6/J, and CD1. Bars, 100 µm. Blood flow is upward. (B) Schematic of VV measurements obtained, overlaid on a P16, left-sided VV. The distance between the commissures (blue line), distance from the center of the tributary to the most proximal commissure (perpendicular to the vessel centerline, red line), and maximum leaflet length (black line) was measured. Tributaries are marked with asterisks (*), and some contain ostial valves. The region shown encompasses the proximal FV and external iliac vein and the VV analyzed lies in the proximal FV. Bar, 100 µm. (C) Number of VVs with and without separated commissures in the strains indicated. *, $P < 0.05$; **, $P < 0.005$; ***, $P < 0.0005$; Fisher's exact test for proportions versus BALB/c. (D and E) Leaflet length (D) and distance from tributary to proximal commissure (E) in SEM images, in the indicated strains, at P6 (green) and P11 (blue). Each marker represents one valve analyzed. **, $P < 0.005$; ***, $P < 0.0005$; ANOVA with Bonferroni correction; post hoc versus BALB/c (at P11).

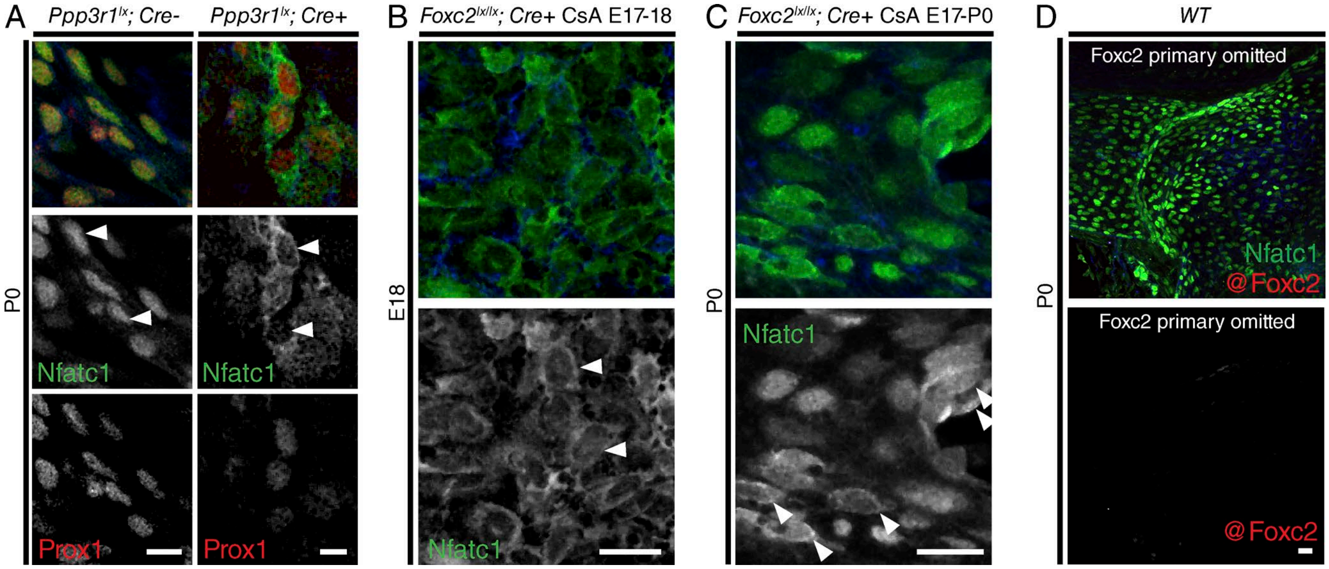

Figure S2. **Regulation of initial VV organization (Cn–Nfat signaling).** (A) Representative images of immunolocalization of Nfatc1 (green) and Prox1 (red) after *Ppp3r1* (CnB) deletion and in littermate controls. Arrowheads indicate (respectively) cytoplasmic and nuclear Nfatc1 localization. *n* = 4 per group. (B and C) Cytoplasmic localization (arrowheads) of Nfatc1 at E18 (B, *n* = 5) and P0 (C) with CsA treatment. *n* = 4. (D) Control for the Foxc2/Nfatc1 colocalization shown in Fig. 3. Anti-Foxc2 primary antibody (raised in sheep) was omitted, demonstrating successful Fab block of cross-reactive binding of anti-sheep secondary to anti-Nfatc1 primary (raised in goat). *n* = 3. (A–D) Blue stain is PECAM1. Bars, 20 µm.

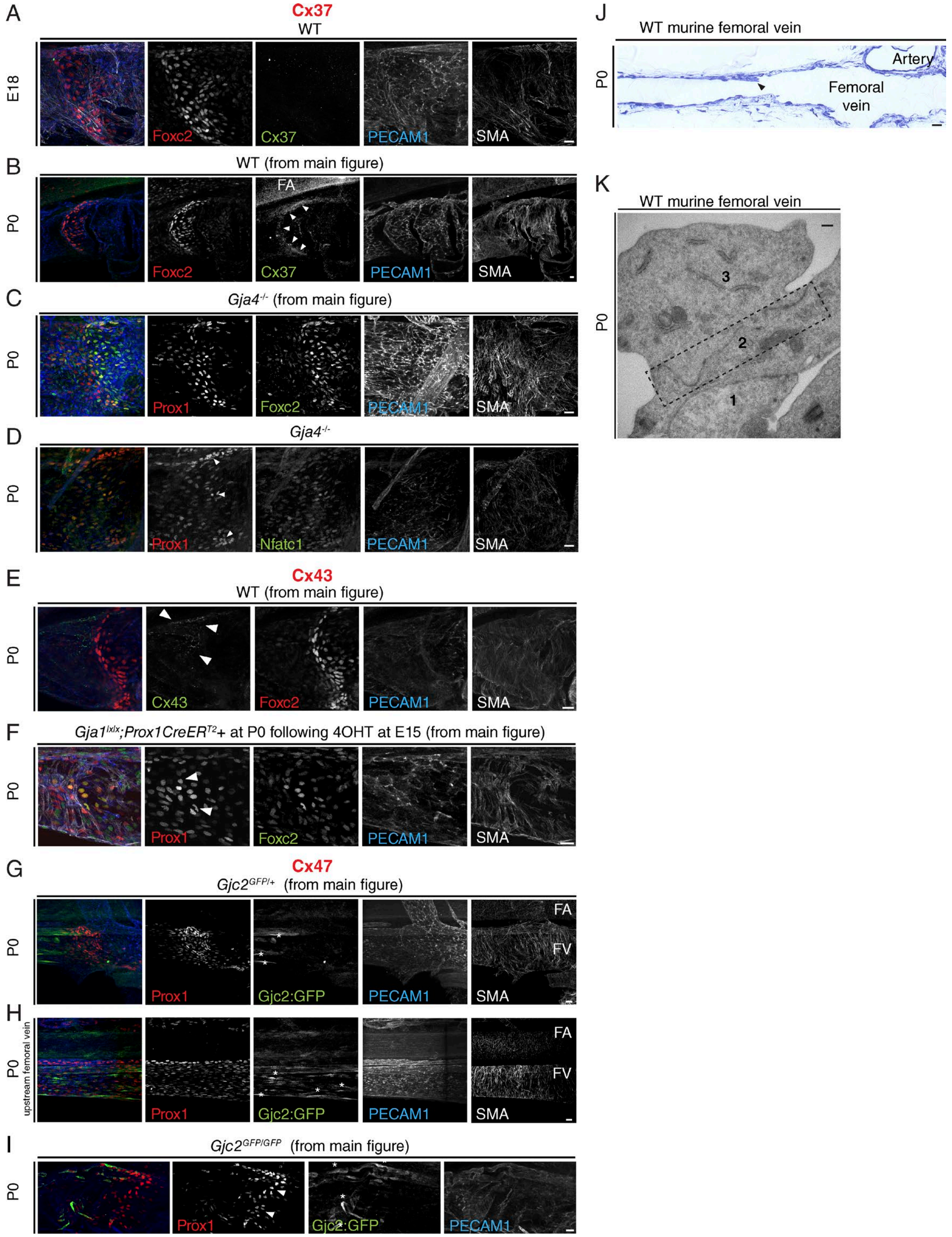

Figure S3. **Regulation of initial VV organization.** (A-I) Multichannel confocal images are shown (along with the individual channels) for the genotypes, ages, and proteins indicated. $n \geq 6$ per condition. Bars, 20 µm. FA, femoral artery. (B) Arrowheads indicate Cx37-expressing VFCs (reproduced from Fig. 4 A). (D) Arrowheads indicate disorganized VFCs. (E) Arrowheads indicate predominant region of Cx43 localization. (F) Arrowheads indicate disorganized VFCs. (G–I) Asterisk (*) indicates GFP (*Gjc2*^{GFP})-expressing cells. The region of the vein just upstream of G is shown in H. (I) Arrowheads indicate VFCs. (J) Tiled micrograph of a semithin section at P0 in which the proximal FV VV (arrowhead) is easily identified as a projection into the lumen, upstream of a tributary. $n = 3$. A major arterial branch runs perpendicular to the vein. The leading edge of the developing VVs was examined by TEM (K), showing three leading-edge VFCs, with the dotted region shown in Fig. 4 G ($n = 3$). Bars: 20 µm (J); 200 nm (K). $n = 3$.

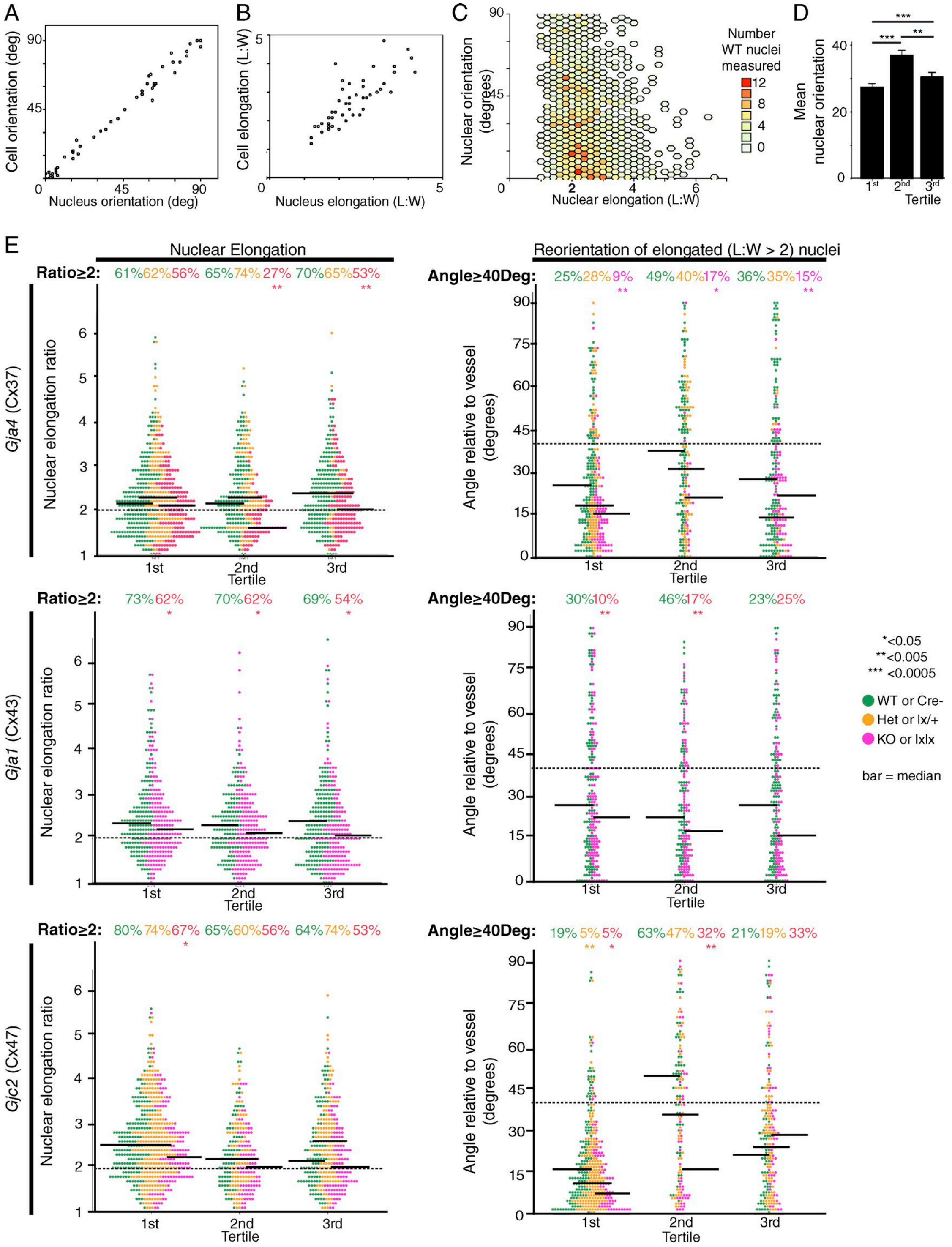

Figure S4. **Quantification of connexin phenotypes.** (A and B) Scatterplots are shown for VFC cellular versus nuclear morphology (orientation in A; elongation in B) in double-transgenic *Prox1CreER*^{T2};*Rosa26*^{mTmG} mice at P0, after induction of Cre at E15. n = 47 cells. (C) A Hex-binned scatterplot is shown for WT VFC nuclear reorientation and elongation. n = 1388 cells. (D) Mean WT VFC nuclear reorientation is shown for each tertile, relating to Fig. 5 A, right. Error bars represent means ± SEM. n = 953 cells, ANOVA with Bonferroni correction. **, P < 0.005; ***, P < 0.0005. (E) Analysis of Prox1^{hi} VFC nuclear reorientation and elongation at P0 with loss of *Gja4*, *Gja1*, or *Gjc2*. Relates to Fig. 5 (C and D). Each data point represents one VFC. n = 6 WT versus 4 *Gja4*$^{+/-}$ versus 8 *Gja4*$^{-/-}$ VVs, 6 WT versus 10 *Gja1*$^{lx/lx}$ VVs, and 5 WT versus 6 *Gjc2*$^{+/-}$ versus 5 *Gjc2*$^{-/-}$ VVs. *, P < 0.05; **, P < 0.005; ***, P < 0.0005. χ^2 test (proportion elongated ≥2, or reoriented ≥40°).

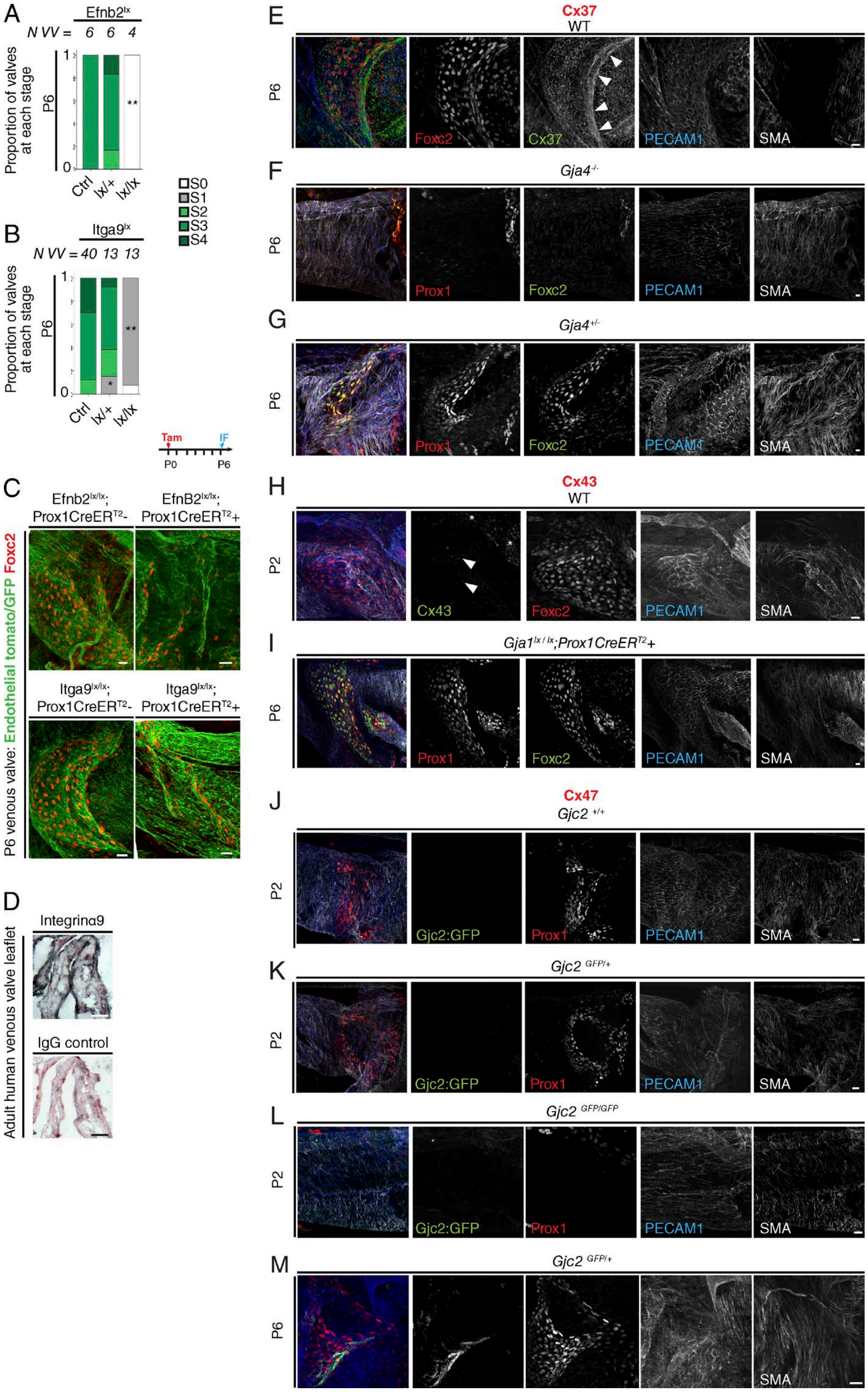

Figure S5. **Maturation of leaflets and commissures.** (A–C) After our change of method to earlier deletion and whole-mount visualization of closed veins, we confirmed the previously described phenotypes after deletion of *Efnb2* (A) and *Itga9* (B) using floxed alleles. The number of VVs analyzed is given above the bars. *, P < 0.05; **, P < 0.005. χ^2 test. (C) Localization of Foxc2 and tomato/GFP (mTmG line) is shown for the indicated genotypes at P6. Bars, 20 µm. (D) Integrinα9 was immunolocalized to human VV leaflets. Bars, 50 µm. (E–G) Multichannel confocal images are shown (along with the individual channels) for the genotypes, ages, and proteins indicated. $n \geq 6$ per condition. Bars, 20 µm. (E) Arrowheads indicate free-edge cells. (H) Arrowheads indicate Cx43, just detectable in VV leaflets (and lymphatics, asterisk [*]) at P2. (J–M) Multichannel confocal images are shown (along with the individual channels) for *Gjc2* expression in the genotypes, ages, and proteins indicated. *Gjc2* expression was examined using the $Gjc2^{GFP}$ reporter with amplification using antibodies raised against GFP. $n = 4$ (J), $n = 3$ (K), $n = 13$ (L), and $n = 4$ (M) VVs. Bars, 20 µm.

Table S1. **Characteristics of study participants**

Mutation	ID	Age	Sex	Genotype	Effect
		yr			
Control	1	24	F		
	2	24	M		
	3	25	M		
	4	30	F		
	5	37	M		
	6	42	F		
	7	42	F		
	8	53	F	Unaffected relatives	
	9	58	F	Unaffected relatives	
	10	69	M	Unaffected relatives	
FOXC2	11	20	M	c.223T>G	Substitution in forkhead domain
	12	47	M	p (Tyr75Asp)	
	13	21	M	c.438G>A	Stop in forkhead domain (after 145 aa)
	14	54	M	p (Trp146Ter)	
	15	22	F	c.595dupC<br>p (His199Profs*264)	Nonsense from aa 199 onward
	16	31	F	c.361C>T<br>p (Arg121Cys)	Substitution in forkhead domain
	17	42	F	c.298C>T<br>p (Gln100Ter)	Stop in forkhead domain (after 99 aa)
	18	51	M	c.595dupC<br>p (His199Profs*264)	Nonsense from aa 199 onward
GJC2	19	17	F	c.143C>T	Substitution in first extracellular loop (Ostergaard et al., 2011; Molica et al., 2014)
	20	46	M	p (Ser48Leu)	
	21	71	M	c.629T>G<br>p (Met210Arg)	Mutation in intracellular loop (Ostergaard et al., 2011)
GJA1	22	44	F	c.617A>G<br>p (Lys206Arg)	Substitution in conserved SRPTEK sequence (Brice et al., 2013)

Participants were matched to the control group by age (P = NS) and sex (P = NS). Controls included nonmutation-carrying relatives of affected individuals.

Table S2. **Number of valves per vein in control group**

Vein	*n* valves per vein in controls			
	n veins	Mean *n* valves	SD	*n* VVs analyzed
Popliteal	16	1.13	0.72	18
Short saphenous	16	3.38	1.45	54
Brachial	18	1.83	1.20	33
Basilic	18	2.39	0.92	43

In control human veins, the number of VVs detected and VV leaflet length followed approximately normal distributions; mean number of VVs and VV length varied significantly by vein (P < 0.0005, ANOVA), and results were subsequently normalized to controls for each vein.

Table S3. **Leaflet lengths per vein in control group**

Vein	Leaflet length in controls		
	n VV measured	Mean VV length	SD
		mm	
Popliteal	14	6.79	1.87
Short saphenous	40	4.00	1.36
Brachial	26	3.88	1.56
Basilic	38	4.93	2.18

REFERENCES

Brice, G., S. Mansour, R. Bell, J.R. Collin, A.H. Child, A.F. Brady, M. Sarfarazi, K.G. Burnand, S. Jeffery, P. Mortimer, and V.A. Murday. 2002. Analysis of the phenotypic abnormalities in lymphoedema-distichiasis syndrome in 74 patients with *FOXC2* mutations or linkage to 16q24. *J. Med. Genet.* 39:478–483. http://dx.doi.org/10.1136/jmg.39.7.478

Molica, F., M.J. Meens, S. Morel, and B.R. Kwak. 2014. Mutations in cardiovascular connexin genes. *Biol. Cell.* 106:269–293. http://dx.doi.org/10.1111/boc.201400038

Ostergaard, P., M.A. Simpson, G. Brice, S. Mansour, F.C. Connell, A. Onoufriadis, A.H. Child, J. Hwang, K. Kalidas, P.S. Mortimer, et al. 2011. Rapid identification of mutations in GJC2 in primary lymphoedema using whole exome sequencing combined with linkage analysis with delineation of the phenotype. *J. Med. Genet.* 48:251–255. http://dx.doi.org/10.1136/jmg.2010.085563
